# Genomic data for 78 chickens from 14 populations

**DOI:** 10.1093/gigascience/gix026

**Published:** 2017-04-18

**Authors:** Diyan Li, Tiandong Che, Binlong Chen, Shilin Tian, Xuming Zhou, Guolong Zhang, Miao Li, Uma Gaur, Yan Li, Majing Luo, Long Zhang, Zhongxian Xu, Xiaoling Zhao, Huadong Yin, Yan Wang, Long Jin, Qianzi Tang, Huailiang Xu, Mingyao Yang, Rongjia Zhou, Ruiqiang Li, Qing Zhu, Mingzhou Li

**Affiliations:** 1 Institute of Animal Genetics and Breeding, College of Animal Science and Technology, Sichuan Agricultural University, Chengdu 611130, China; 2 Novogene Bioinformatics Institute, Beijing 100083, China; 3 Division of Genetics, Department of Medicine, Brigham and Women's Hospital, Harvard Medical School, Boston, MA 02115, USA; 4 Department of Animal Science, Oklahoma State University, Stillwater, OK 74078, USA; 5 Hubei Key Laboratory of Cell Homeostasis, Laboratory of Molecular and Developmental Genetics, College of Life Sciences, Wuhan University, Wuhan 430072, China.

**Keywords:** chicken, genetic diversity, population genomics, whole genome resequencing

## Abstract

**Background:** Since the domestication of the red jungle fowls (*Gallus gallus*; dating back to ∼10 000 B.P.) in Asia, domestic chickens (*Gallus gallus domesticus*) have been subjected to the combined effects of natural selection and human-driven artificial selection; this has resulted in marked phenotypic diversity in a number of traits, including behavior, body composition, egg production, and skin color. Population genomic variations through diversifying selection have not been fully investigated. **Findings:** The whole genomes of 78 domestic chickens were sequenced to an average of 18-fold coverage for each bird. By combining this data with publicly available genomes of five wild red jungle fowls and eight Xishuangbanna game fowls, we conducted a comprehensive comparative genomics analysis of 91 chickens from 17 populations. After aligning ∼21.30 gigabases (Gb) of high-quality data from each individual to the reference chicken genome, we identified ∼6.44 million (M) single nucleotide polymorphisms (SNPs) for each population. These SNPs included 1.10 M novel SNPs in 17 populations that were absent in the current chicken dbSNP (Build 145) entries. **Conclusions:** The current data is important for population genetics and further studies in chickens and will serve as a valuable resource for investigating diversifying selection and candidate genes for selective breeding in chickens.

## Introduction

### Genome sequencing and sequence filtering

**Figure 1:**
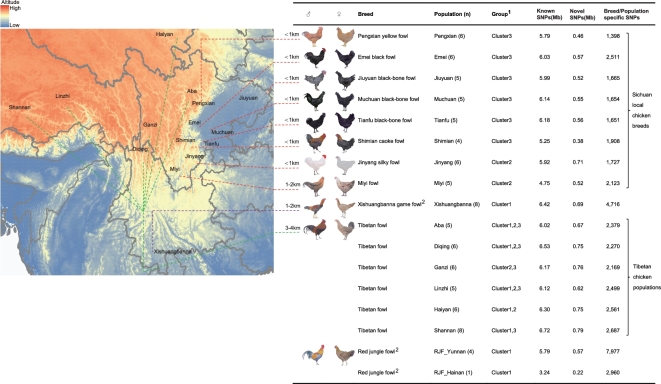
Sample information and comparison of identified SNPs in each breed/population with the chicken variants database (dbSNP, Build 145). Known SNPs are SNPs already in chicken dbSNP. The map displayed here is the geographic distribution of domestic chicken populations; **numbers above the dashed lines** are altitudes. **Red and green localities** represent eight lowland and six highland chicken populations respectively, sampled in this study. ^a^Individual distribution to each group can be found in Additional File 1: Table S1. ^b^The whole genome sequencing data of eight game fowls and five RJFs were downloaded from the NCBI.

The 78 blood samples (36 Tibetan fowls from the Qinghai-Tibet Plateau and 42 domestic fowls from Szechwan Basin) (Fig. 1) were collected from the wing vein. The animal handling experiments were approved by the Institutional Animal Care and Use Committee of Sichuan Agricultural University under permit number YCS-B20100804. Genomic DNA was extracted from these samples following standard procedures. In total, we generated ∼1.69 trillion bases of resequencing data of the whole genomes from 78 birds (18.03-fold coverage for each individual) on the Illumina Hiseq 2500 platform (Additional File 1: Table S1). In addition, previously published genome sequence data from five red jungle fowls (RJF) and eight Xishuangbanna game fowls (∼16.6-fold coverage for each individual) were downloaded and analyzed (GenBank accession number PRJNA241474) (Fig. 1).

We also filtered out the adapter sequences (>10 nt aligned to the adapter, allowing ≤10% mismatches), low-quality reads (i.e., ≥10% unidentified nucleotides or >50% bases having Phred quality <5) and duplicated reads generated in the library construction process.

## Data Analysis

### Reads mapping

The high-quality paired-end reads were mapped to the reference chicken genome (Galgal4.78) using Burrows-Wheeler Aligner (BWA) software (version 0.7.8) [1] with the command “mem -t 10 -k 32,” and BAM alignment files were generated using SAMtools (version 0.1.19) [2].

Next, we improved the alignment results using the following steps:

- The aligned reads with mismatches ≥5 or mapping quality = 0 were removed.
- The alignment results were then corrected using Picard (version 1.96; http://broadinstitute.github.io/picard/) with two core commands. The “AddOrReplaceReadGroups” command was used to replace all read groups in the INPUT file with a new read group and assign all reads to this group in the OUTPUT BAM. The “FixMateInformation” command was used to ensure that all mate-pair information was in sync between each read and its mate pair.
- Removed potential Polymerase Chain Reaction (PCR) duplications. If multiple read pairs had identical external coordinates, only the pair with the highest mapping quality was retained.
- Realigned reads around the insertions and deletions (InDels). We downloaded variants registered in chicken dbSNP database (Build 145) from NCBI and generated a target list of intervals by using the command “RealignerTargetCreator” in package Genome Analysis Toolkit (GATK; version 3.1-1- g07a4bf8) [3]. We further used the command “IndelRealigner” to identify regions for realignment where at least one read contains a registered InDel with a cluster of mismatching bases around it.

Consequently, ∼21.30 Gb of high-quality data of each individual mapping to reference chicken genome (Additional File 1: Table S1) were used for subsequent analysis.

### Single nucleotide polymorphism calling

We first detected individual single nucleotide polymorphisms (SNPs) simultaneously confirmed by both SAMtools and GATK. The highly accurate alignment was processed using the “mpileup” program in SAMtools with the parameters “-C 50 -D -S -m 2 -F 0.002 -d 1000” (“-C 50” is a recommended parameter, “-D” and “-S” are default parameters, “-m 2,” “-F 0.002,” and “-d 1000” are required parameters). The variants were then filtered for downstream analysis by requiring a coverage ranging from 4 to 200, a minimum root-mean-square mapping quality of 20, and no gaps present within a 3 bp window. Meanwhile, we detected genomic variants for each bird using GATK with the HaplotypeCaller-based method; before calling variants, the base quality scores were recalibrated using the command “BaseRecalibrator,” which provides empirically accurate base quality scores for each base in every read. After SNP calling, we applied hard filter command “VariantFiltration” to exclude potential false-positive variant calls with the parameter “–filterExpression ‘QD < 10.0 ‖ FS > 60.0 ‖ MQ < 40.0 ‖ ReadPosRankSum< −8.0’ -G_filter ‘GQ<20.’” As a result, ∼6.44 Mb SNPs for each breed/population were identified (Additional File 1: Table S2).

Then we merged all individual SNPs into a population SNP matrix. Finally, we obtained 8.53 Mb of highly credible SNPs after using strict criteria with filtering minor allele frequency (MAF) < 0.05 and missing genotype > 10% in the chicken population. Subsequently, the package ANNOVAR (version 20 May 2013) [4] was used to annotate SNPs causing nonsense and missense mutations.

### Insertions and deletions calling

The candidate InDels were called along with SNPs by GATK for 91 individuals. We first sifted structural variations for each sample by GATK with the SelectVariants-based method. Then, we applied hard filter command “VariantFiltration” to exclude potential false-positive variant calls with the parameter “–filterExpression ‘QD < 2.0 ‖ FS > 200.0 ‖ ReadPosRankSum < −8.0 ‖ InbreedingCoeff< −0.8.’” Finally, we only retained the 1–30 bp InDels for downstream analysis.

### Analysis of the population structure and evolutionary history

Rooted neighbor-joining phylogenetic tree was constructed under the p-distances model in TreeBeST (version 1.9.2; http://treesoft.sourceforge.net/treebest.shtml) using Japanese quail as an outgroup. The reliability of each branch was evaluated by bootstrapping [5] with 1000 replicates. The phylogenetic relationships of the individual genomes were also estimated using principle component analysis (PCA) with the population-scale SNPs using the EIGENSOFT (version 5.0) [6] software, and the eigenvectors were obtained from the covariance matrix generated by R function reigen.

## Findings

### Genetic diversity

A total of 7.43 Mb of SNPs out of 8.53 Mb highly credible SNPs were already present in the chicken dbSNP database (known SNPs), and 1.10 Mb SNPs were assigned as novel ones. All 1.10 Mb novel SNPs have been submitted to dbSNP (accession numbers from ss2585830405 to ss2586846514 and ss2137077162; see Additional File 2). We further conducted a comparative genomics analysis of 91 chickens from 15 domestic and two wild populations (Fig. 1). The general phenotypic differences between red jungle fowls (RJF), Tibetan fowls, and Sichuan local fowls are shown in Additional File 1: Table S3. We identified 3.46–7.52 Mb SNPs for each breed/population that were confirmed by both SAMtools and GATK softwares (Additional File 1: Table S2). There were 1398 to 7977 SNPs specifically detected in a breed/population (Fig. 1). Nucleotide variability (θπ) and polymorphism (θω) in each population were analyzed using the method of sequence diversity statistics [7]. Compared with Sichuan local chicken breeds (θπ = 2.35 × 10^−3^ and θω = 2.13 × 10^−3^), Tibetan chicken populations have relatively higher genetic diversity (θπ *=* 2.58 × 10^−3^, *P <* 2.2 × 10^−16^ and θω *=* 2.35 × 10^−3^, *P =* 0.656, Mann-Whitney U test) (Additional File 1: Fig. S1).

As shown in Additional File 1: Fig. S2, although most novel SNPs (89.02%) had a low allele frequency (<0.2 of 91 individuals) compared with the known SNPs (44.02%), only 9918 (0.88% of 1.10 M) novel SNPs were specifically detected in one breed/population (at least in one individual). These novel SNPs exhibited similar read depth with the known SNPs (median of 20× versus 19×), which are both comparable with the average depth for the genome (median of 1.14-fold versus 1.06-fold) (Additional File 1: Fig. S3). In addition, we observed that more than 75% of the novel SNPs and 86% of the known SNPs were in non-repeat regions. These results suggest that the novel SNPs will serve as a potentially valuable resource for further chicken studies.

Overall distribution of the lengths of insertions and deletions showed that more than 80% of the InDels were 1–5 bp in length (Additional File 1: Fig. S4). Repetitive elements (10.61% of the genome and containing ∼15.70% of InDels) are an important source of structural variation in the chicken genome (Additional File 1: Fig. S5). About a half of InDels (48.39% to 51.52%) occurred in the intergenic regions (588.65 Mb and 56.23% of the genome). The introns (403.35 Mb and containing ∼43.86% of InDels) showed higher incidence of InDels than the coding sequences (25.81 Mb and containing ∼1.77% of InDels) (Additional File 1: Fig. S6). We observed an enrichment of short InDels (1–15 bp in length) in coding sequences that were multiples of 3 bp compared to whole genome sequences, which is expected to preserve the reading frame (Additional File 1: Fig. S7).

### Population genetics

**Figure 2:**
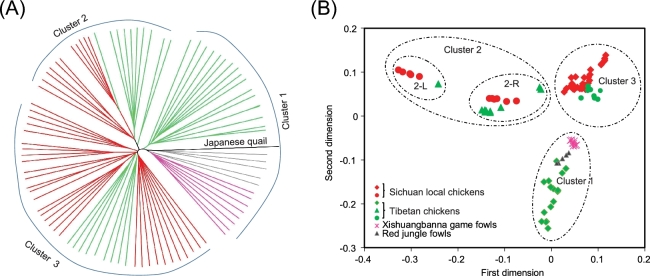
Population genetics of studied chickens. **A)** Rooted neighbor-joining phylogenetic tree with the Japanese quail as an outgroup. The reliability of each branch was evaluated by bootstrapping with 1000 replicates. Different groups of chicken populations: Sichuan local chickens (**red**), Tibetan chickens (**green**), Xishuangbanna game fowls (**purple**), RJFs (**gray**), and Japanese quail (**black**). **B)** Principal component plots. The first dimension and second dimension are shown. The fraction of the variance explained was 8.91% for eigenvector 1 (*P* < 0.05, Tracy-Widom test) and 7.43% for eigenvector 2 (*P* < 0.05, Tracy-Widom test).

The neighbor-joining phylogenetic tree revealed the segregation of 15 domestic populations and two wild RJF populations into three distinct clusters (cluster 1, cluster 2, and cluster 3) (Fig. 2A). The principal component analysis (PCA) as implemented in EIGENSOFT package [6] recapitulated these findings (Fig. 2B) and revealed that cluster 2 can be further split into two sub-clusters. The Tibetan fowls in cluster 2 are genetically closer to Jinyang silky fowls (sub-cluster 2-R) than Miyi fowls (sub-cluster 2-L) (Fig. 2B). Different from a previous report on the two independent origins of Tibetan chickens [8], we revealed the presence of at least three distinct clusters among the six geographically representative populations of Tibetan fowls: the fowls inhabiting Tibet and Qinghai (in cluster 1) were genetically closer to RJF, while the Tibetan chickens inhabiting Yunnan and Sichuan (clusters 2 and 3) were closer to the domestic populations (Fig. 1). These distinct distribution patterns and expansion signatures suggested that the divergent Tibetan clades may have originated from different regions, such as Yunnan, southwest China, and/or surrounding areas [8]. We found that many Tibetan chickens clustered with other Sichuan local chicken breeds in cluster 2 and cluster 3, which may be attributable to shared ancestral polymorphism and/or recent introgression events by way of possible crossbreeding between Tibetan chickens with the geographically neighboring Sichuan local chickens. Although this inference is consistent with recent breeding activities in the Tibet plateau [8], further analysis is required to explore the introgression between them.

## Conclusions

Understanding the nature of diversifying selection, especially detecting selection signatures, and identifying genes in a genome that are, or have been, under selection have been the hot topics of interest. This study provides a comparative genomic landscape of variations in 17 chicken populations to understand genetic variations underlying the phenotypic diversity of chicken breeds/populations. These data will serve as a valuable resource for investigating diversifying selection and candidate genes for selective breeding in chickens.

## Funding

This work was supported by grants from the China Agricultural Research System (CARS-41), the 12th Five Year Plan for Breeding Program in Sichuan–Selective breeding of new breeds and the synthetic strains in laying hens (2011NZ0099-7), the National Natural Science Foundation of China (31402063 and 31522055), the Sichuan Provincial Department of Science and Technology Program (2015JQO023), the National Program for Support of Top-notch Young Professionals, and the Young Scholars of the Yangtze River.

## Availability of supporting data

The sequencing data for this project have been deposited in the NCBI sequence read archive (SRA) under accession number SRP067615. Additional data, including sequence variations in variant call format, are available in the *GigaScience* repository, GigaDB [9]. All supplementary figures and tables are provided in Additional File 1:

Table S1. A summary of the chickens used in this study: regions of collection/population and sequencing depths.

Table S2. SNP annotation and genetic diversity of the 17 chicken populations analyzed in this study.

Table S3. The general phenotypic differences between red jungle fowls, Tibetan chickens, and Sichuan local chickens.

Figure S1. Average nucleotide polymorphism (*θ_w_*) and nucleotide diversity (*θ_π_*) among Sichuan local chickens, Tibetan chickens, and red jungle fowls.

Figure S2. Allele frequency spectra in 91 birds and number of alleles distribute in 1 to 17 chicken breeds/populations.

Figure S3. Comparison of read depth between the known and novel SNPs.

Figure S4. Overall distribution of the lengths of InDels (1–30 bp).

Figure S5. Percentage composition of InDels in repeat elements.

Figure S6. Percentage distribution (A) and probability (B) for InDels across different genomic elements.

Figure S7. Length distribution of small InDels in the whole genome (A) and coding sequence (CDS) regions (B).

Additional File 2 includes Accession numbers of 1.10 Mb novel SNPs (txt 20.3 Mb).

## Conflicts of interest

The authors declare no competing financial interests.

## Authors’ contributions

Q.Z., and M.Z.L. designed and supervised the project. B.C., M.L., H.Y., Y.W., X.Z., G.Z., U.G., M.J.L., L.Z., M.Y., R.J., R.L., and X.Z. collected and generated the data and performed the preliminary bioinformatic analyses. T.C., S.T., Y.L., Z.X., L.J., Q.T., H.X., and X.Z. filtered the data and performed the majority of the population genetic analyses. D.L. and T.C. wrote the manuscript.

## Supplementary Material

GIGA-D-16-00092_Original_Submission.pdf

GIGA-D-16-00092_Revision_1.pdf

GIGA-D-16-00092_Revision_2.pdf

GIGA-D-16-00092_Revision_3.pdf

Response_to_reviewer_comments_Revision_2.pdf

Response_to_reviewer_comment_Revision_1.pdf

Reviewer_1_Report_(Original_Submission).pdf

Reviewer_1_Report_(Revision_1).pdf

reviewer_1_report_(Revision_2).pdf

Reviewer_2_Report_(Original_Submission).pdf

Reviewer_2_Report_(Revision_1).pdf

Reviewer_3_Report_(Original_Submission).pdf

Reviewer_3_Report_(Revision_1).pdf

Supplemental material The sequencing data for this project have been deposited in the NCBI sequence read archive (SRA) under accession number SRP067615. Additional data, including sequence variations in variant call format, are available in the *GigaScience* repository, GigaDB [9]. All supplementary figures and tables are provided in Additional File 1:
Table S1. A summary of the chickens used in this study: regions of collection/population and sequencing depths.
Table S2. SNP annotation and genetic diversity of the 17 chicken populations analyzed in this study.
Table S3. The general phenotypic differences between red jungle fowls, Tibetan chickens, and Sichuan local chickens.
Figure S1. Average nucleotide polymorphism (*θ_w_*) and nucleotide diversity (*θ_π_*) among Sichuan local chickens, Tibetan chickens, and red jungle fowls.
Figure S2. Allele frequency spectra in 91 birds and number of alleles distribute in 1 to 17 chicken breeds/populations.
Figure S3. Comparison of read depth between the known and novel SNPs.
Figure S4. Overall distribution of the lengths of InDels (1–30 bp).
Figure S5. Percentage composition of InDels in repeat elements.
Figure S6. Percentage distribution (A) and probability (B) for InDels across different genomic elements.
Figure S7. Length distribution of small InDels in the whole genome (A) and coding sequence (CDS) regions (B).
Additional File 2 includes Accession numbers of 1.10 Mb novel SNPs (txt 20.3 Mb).
