# Supplementary material for: Genomic data for 78 chickens from 14 populations
Source: Gigascience. 2017 Apr 18;6(6):1–5. doi: 10.1093/gigascience/gix026 (PMC5449643; doi:10.1093/gigascience/gix026)
Supplement: GIGA-D-16-00092_Original_Submission.pdf [file gix026_GIGA-D-16-00092_Original_Submission.pdf]

Manuscript Click here to download Manuscript Gigascience.docx
Click here to view linked References

The genomics data for 78 chickens from 15 populations

Diyan Li[1†], Tiandong Che[1†], Binlong Chen[1†], Shilin Tian[1,2†], Xuming Zhou[3†], Guolong Zhang[4†], Miao Li[1], Xiaoling Zhao[1], Huadong Yin[1], Yan Wang[1], Ruiqiang Li[2], Qing Zhu[1] and Mingzhou Li[1]

[1] Institute of Animal Genetics and Breeding, College of Animal Science and Technology, Sichuan Agricultural University, Chengdu, China
[2] Beijing Novogene bioinformatics Technology Co., Ltd, Beijing, China
[3] Division of Genetics, Department of Medicine, Brigham and Women's Hospital, Harvard Medical School, Boston, USA
[4] Department of Animal Science, Oklahoma State University, Stillwater, Oklahoma, USA

[†] These authors contributed equally to this work.
Correspondence: zhuqingsicau@163.com; mingzhou.li@sicau.edu.cn.

Abstract

Background: Since the domestication of the red jungle fowl (*Gallus gallus*) (dating back to ~10,000 B.P.) in Asia, domestic chickens (*Gallus gallus domesticus*) have been subjected to the combined effects of natural selection and human-driven artificial selection; this has resulted in marked phenotypic diversity in a number of traits, including behavior, body composition, egg production and skin color. Population genome variations through diversifying selection have not been fully reported.

Findings: The whole genomes of 78 domestic chickens were sequenced to an average of approximately 18-fold coverage depth for each individual with a total of 1.69T reads. Together with the available genomes of 5 wild red jungle fowl and 8 Xishuangbanna game fowls, we conducted a comparative genomics analysis of 91 individuals from 16 populations. After aligning approximately 21.9-Gb high-quality reads of each individual to the reference genome, we identified 5.27-6.65 million SNPs for each population. These SNPs included 1.1 million novel SNPs in 15 populations that were absent from the current chicken dbSNP database. These data are abundant resources for poultry breeding.

Conclusions: The data will serve as a valuable resource for investigating diversifying selection and candidate genes for selective breeding in chicken.

Keywords: Chicken, Genome diversity, Population genomics, Next generation sequencing

Data description

Genome sequencing

The 78 blood samples were collected from the wing vein, this experimentation was approved by the Institutional Animal Care and Use Committee of Sichuan Agricultural University under permit number YCS-B20100804. Genomic DNA was extracted from these samples using phenol following standard procedures. The whole genomes of 36 Tibetan fowls from the Qinghai-Tibet Plateau and 42 domestic fowls from Szechwan Basin (**Figure 1 and Additional file 1: Table S1**) were sequenced on the Illumina Hiseq 2000 platform. The raw reads were cleaned by removing low-quality paired reads, which mainly resulted from base-calling

duplicates and adapter contamination. Reads with ≥10% of unidentified nucleotides, >10 nt aligned to the adapter, allowing ≤10% mismatches, >50% of bases with Phred quality <33, and putative PCR duplicates generated in the library construction process were removed. In addition, previously published genome sequence data from two other populations (red jungle fowls and Xishuangbanna game fowls) (GenBank accession number PRJNA241474) were downloaded and analyzed.

Data generation and analysis

Read mapping

The high-quality paired-end reads were mapped to the reference chicken genome [1] (Galgal4.78) using BWA software [2] with the command 'mem -t 10 -k 32'. Then, BAM alignment files were generated using SAMtools [3]. Next, we improved the alignment results by filtering the alignment reads with mismatches ≤5 and mapping quality=0. The alignment results were then corrected using Picard (http://sourceforge.net/projects/picard/) with two core commands. The 'AddOrReplaceReadGroups' command was used to replace all read groups in the INPUT file with a new read group and assign all reads to this read group in the OUTPUT BAM. The 'FixMateInformation' command was used to ensure that all mate-pair information was in sync between each read and its mate pair. If multiple read pairs had identical external coordinates, only the pair with the highest mapping quality was retained. Reads were realigned around the indels by first identifying the regions for realignment where at least one read contains an indel with a cluster of mismatching bases around it.

SNP calling

To identify high-credibility variation in the 91 chickens, the highest-accuracy alignment was first processed using the 'mpileup' program in SAMtools with the parameters '-C -D -S -m 2 -F 0.002 -d 1000' [3]. The variants were filtered for downstream analysis by requiring a minimum coverage of 4 and a maximum coverage of 200, a minimum RMS mapping quality of 20 and no gaps present within a 3-bp window. We then detected genomic variants for each chicken breed using the Genome Analysis Toolkit (GATK, version v3.1) [4] with the HaplotypeCaller-based method. Before calling variants, the base quality scores were recalibrated using GATK, which provides empirically accurate base quality scores for each base in every read. After SNP calling, we applied variant quality recalibration to exclude potential false-positive variant calls. We used the command 'VariantFiltration' with the parameter '--filterExpression "QD < 10.0 || FS > 60.0 || MQ < 40.0 || ReadPosRankSum < -8.0" -G_filter "GQ<20"'. The package ANNOVAR[5] was used to annotate SNPs causing nonsense and missense mutations.

Analysis of the population structure and evolutionary history

The phylogenetic relationships of the individual genomes were estimated using principle component analysis (PCA) with the population-scale SNPs using the EIGENSOFT4.2 [6]

software package, and the eigenvectors were obtained from the covariance matrix using the R function reigen.

Findings

The whole genomes of 78 domestic chickens were sequenced to an average of approximately 18-fold coverage depth for each individual with a total of 1.69T reads (**Additional file 1: Table S1**). The general phenotypic differences between RJF, Tibetan and Sichuan local chicken breeds were presented in **Additional file 1: Table S2**. Together with the available genomes of 5 wild red jungle fowl and 8 Xishuangbanna game fowls (approximately 16.6-fold coverage for each individual), we conducted a comparative genomics analysis of 91 individuals from 15 domestic populations and an ancestry population. After aligning approximately 21.9-Gb high-quality reads of each individual to the reference genome assembly (Galgal4.78) using the Burrows-Wheeler Aligner (BWA) tool [2], we performed population single nucleotide polymorphism (SNP) calling using both SAMtools and the Genome Analysis Toolkit (GATK). We identified 5.27-6.65 million SNPs for each population that were confirmed by both softwares. These SNPs included 1.1 million novel SNPs in 15 populations that were absent from the current chicken dbSNP database (Build 145) (**Figure 1**).

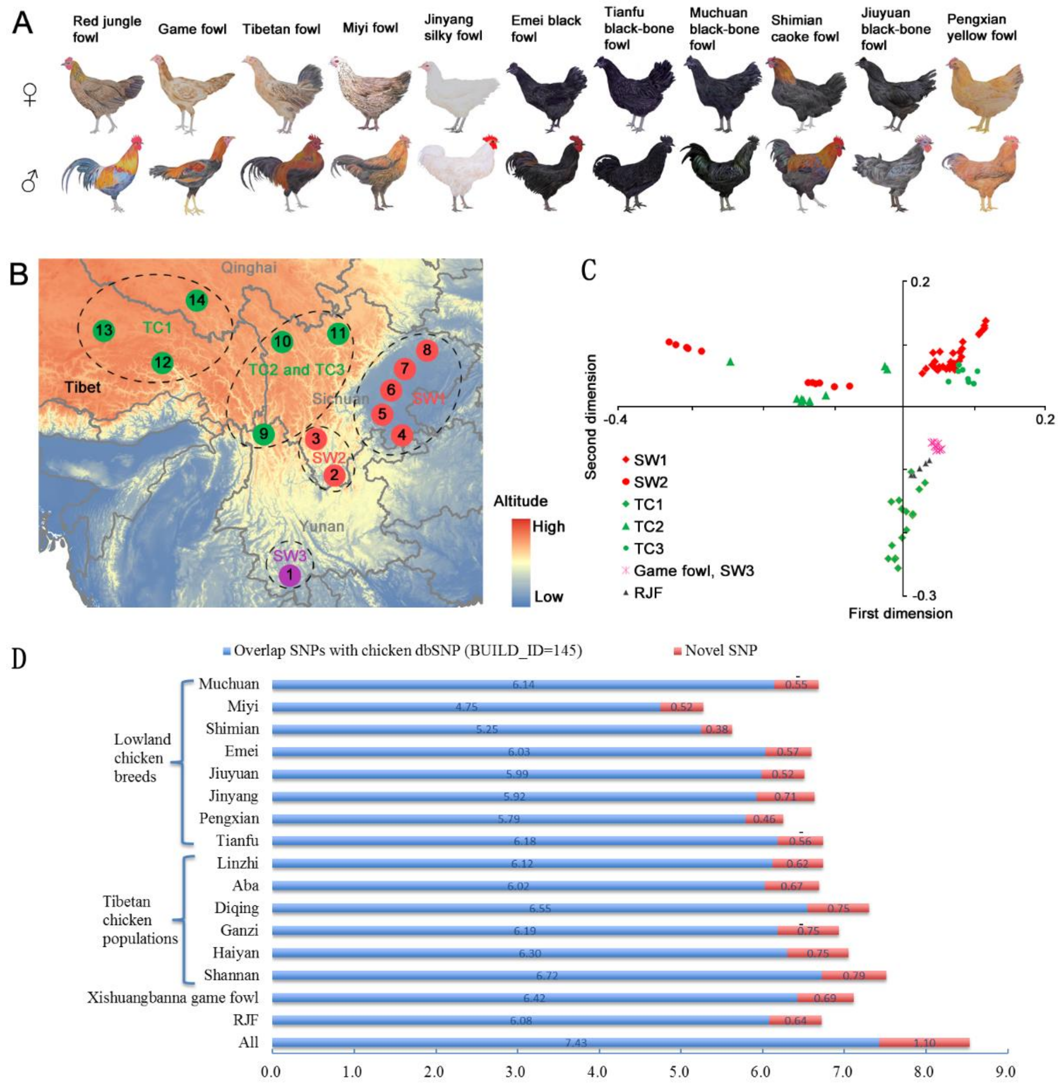

Figure 1 Phenotypic differences, map of sampling localities, population structure and SNPs of the 91 chickens. (A**)** The striking phenotypic differences among the nine domestic chicken breeds and red jungle fowls (RJFs) analyzed in this study. (B) Geographic distribution of the chicken populations. Red and green localities represent eight lowland and six highland chicken populations used in this study, respectively (1-14 represent Xishuangbanna, Miyi, Jinyang, Muchuan, Emei, Jiuyuan, Chengdu, Pengxian, Diqing, Ganzi, Aba, Shannan, Linzhi and Haiyan, respectively). (C) Principal component plots. The first dimension and second dimension are shown. The fraction of the variance explained was 8.91% for eigenvector 1 ($P<0.05$, Tracy-Widom test) and 7.43% for eigenvector 2 ($P<0.05$, Tracy-Widom test). (D) Comparison of identified SNPs in the 15 chicken populations and red jungle fowl with the public database of chicken variants (dbSNP, build 145).

Nucleotide polymorphism and diversity in each population were analyzed using the method of sequence diversity statistics (θ_π and θ_ω) [7]. We observed a relatively higher genetic diversity in all 6 TC sampling locations (pairwise nucleotide diversity, θ_π =2.63 $\times10^{-3}$, and average nucleotide polymorphism θ_ω = 2.02 $\times10^{-3}$) compared to the other domestic populations (θ_π = 2.43 $\times10^{-3}$ and θ_ω = 2.03 $\times10^{-3}$). These values were comparable to those of the RJFs (θ_π = 2.58 $\times10^{-3}$ and θ_ω = 2.38 $\times10^{-3}$) (**Additional file 1: Table S3**). Particularly, more than half of the SNPs were detected in intergenic regions in each population, suggesting that changes at regulatory sites may have played a prominent role in diversifying selection of various chicken breeds.

Phylogenetic analysis based on genome-wide SNPs using the neighbor-joining (NJ) method revealed the segregation of 15 domestic populations and wild RJFs into seven distinct lineages (SW3, SW2, SW1, TC1, TC2, TC3 and a RJF group (**Figure 1**). A similar pattern of clustering was also observed based on principal component analysis (PCA) using EIGENSOFT4.2 [6]. These distinct distribution patterns and expansion signatures suggest that the divergent Tibetan clades may have originated from different regions, such as Yunnan, southwest China and/or surrounding areas [8].

Availability of supporting data

The sequencing data for this project have been deposited in the NCBI sequence read archive (SRA) under accession number SRP067615. All supplementary figures and tables are provided in Additional file 1.

Additional file

Additional file 1: Table S1, Table S2 and Table S3. (xlsx 24KB)

Funding

This work was supported by China Agricultural Research System (CARS-41), the 12th Five Year Plan for breeding program in Sichuan-Selective breeding of new breeds and the synthetic strains in laying hens (2011NZ0099-7), National Natural Science Foundation of China (31160432) and Sichuan Provincial Department of Science and Technology Program (2015JQO023).

Authors' contributions

Q.Z., and M.L. designed and supervised the project. B.C., M.L., H.Y., Y.W., X.Z., G.Z. and X.Z collected and generated the data, and performed the preliminary bioinformatic analyses. S.T., filtered the data and performed the majority of the population genetic analysis. D.L. and T.C. wrote the manuscript.

Competing financial interests

The authors declare no competing financial interests.

References

1. Hillier LW, Miller W, Birney E, Groenen MAM, Crooijmans RPMA, Aerts J, et al. Sequence and comparative analysis of the chicken genome provide unique perspectives on vertebrate evolution. Nature. 2004; 432: 695-716.
2. Li H,Durbin R. Fast and accurate long-read alignment with Burrows–Wheeler transform. Bioinformatics. 2010; 26: 589-595.
3. Li H, Handsaker B, Wysoker A, Fennell T, Ruan J, Homer N, et al. The sequence alignment/map format and SAMtools. Bioinformatics. 2009; 25: 2078-2079.
4. McKenna A, Hanna M, Banks E, Sivachenko A, Cibulskis K, Kernytsky A, et al. The Genome Analysis Toolkit: a MapReduce framework for analyzing next-generation DNA sequencing data. Genome Res. 2010; 20: 1297-1303.

5. Conesa A, Götz S, García-Gómez JM, Terol J, Talón M,Robles M. Blast2GO: a universal tool for annotation, visualization and analysis in functional genomics research. Bioinformatics. 2005; 21: 3674-3676.
6. Nick P, Price AL,David R. Population structure and eigenanalysis. Plos Genetics. 2006; 2: 2074-2093.
7. Nei M, .,Li WH. Mathematical model for studying genetic variation in terms of restriction endonucleases. Proc Natl Acad Sci U S A. 1979; 76: 5269-5273.
8. Ming-Shan W, Yan L, Min-Sheng P, Li Z, Zong-Ji W, Qi-Ye L, et al. Genomic Analyses Reveal Potential Independent Adaptation to High Altitude in Tibetan Chickens. Molecular Biology & Evolution. 2015; 32: 1880-9.

Click here to access/download
Supplementary Material
Aditional file 1.xlsx
