# Supplementary material for: Genomic data for 78 chickens from 14 populations
Source: Gigascience. 2017 Apr 18;6(6):1–5. doi: 10.1093/gigascience/gix026 (PMC5449643; doi:10.1093/gigascience/gix026)
Supplement: GIGA-D-16-00092_Revision_1.pdf [file gix026_GIGA-D-16-00092_Revision_1.pdf]

Manuscript Click here to download Manuscript Gigascience-revised-11-8.docx

Genomic data for 78 chickens from 14 populations

Diyan Li[1†], Tiandong Che[1†], Binlong Chen[1†], Shilin Tian[1,2†], Xuming Zhou[3†], Guolong Zhang[4†], Miao Li[1], Uma Gaur[1], Majing Luo[5], Long Zhang[1], Zhongxian Xu[1], Xiaoling Zhao[1], Huadong Yin[1], Yan Wang[1], Long Jin[1], Qianzi Tang[1], Huailiang Xu[1], Mingyao Yang[1], Rongjia Zhou[5], Ruiqiang Li[2], Qing Zhu[1] and Mingzhou Li[1]

[1] Institute of Animal Genetics and Breeding, College of Animal Science and Technology, Sichuan Agricultural University, Chengdu, China
[2] Beijing Novogene bioinformatics Technology Co., Ltd, Beijing, China
[3] Division of Genetics, Department of Medicine, Brigham and Women's Hospital, Harvard Medical School, Boston, USA
[4] Department of Animal Science, Oklahoma State University, Stillwater, Oklahoma, USA
[5] Department of Genetics, College of Life Sciences, Wuhan University, Wuhan, China
[†] These authors contributed equally to this work.
Correspondence: zhuqingsicau@163.com; mingzhou.li@sicau.edu.cn.

Abstract

Background: Since the domestication of the red jungle fowls (*Gallus gallus*) (dating back to ~10,000 B.P.) in Asia, domestic chickens (*Gallus gallus domesticus*) have been subjected to the combined effects of natural selection and human-driven artificial selection; this has resulted in marked phenotypic diversity in a number of traits, including behavior, body composition, egg production and skin color. Population genomic variations through diversifying selection have not been fully reported.

Findings: The whole genomes of 78 domestic chickens were sequenced to an average of 18-fold coverage for each bird. By combining this data with publicly available genomes of 5 wild red jungle fowls and 8 Xishuangbanna game fowls, we conducted a comprehensive comparative genomics analysis of 91 chickens from 17 populations. After aligning ~21.30 gigabase (Gb) high quality data of each individual to the reference chicken genome, we identified ~6.44 million (M) SNPs for each population. These SNPs included 1.10 M novel SNPs in 17 populations that were absent in the current chicken dbSNP (Build 145) entries.

Conclusions: The current data is important for population genetics and further studies in chicken, and will serve as a valuable resource for investigating diversifying selection and candidate genes for selective breeding in chicken.

Keywords: Chicken, Genetic diversity, Population genomics, Whole-genome resequencing

Data description

Genome sequencing and sequence filtering

The 78 blood samples (36 Tibetan fowls from the Qinghai-Tibet Plateau and 42 domestic fowls from Szechwan Basin) (Table 1, Figure 1A and B) were collected from the wing vein. The animal handling experiments were approved by the Institutional Animal Care and Use Committee of Sichuan Agricultural University under permit number YCS-B20100804. Genomic DNA was extracted from these samples following standard procedures. In total, we generated ~1.69 trillion base resequencing data of the whole genomes from 78 birds (18.03-fold coverage for each individual) on the Illumina Hiseq 2500 platform. In addition, previously

published genome sequence data from 5 red jungle fowls (RJF) and 8 Xishuangbanna game fowls (~16.6-fold coverage for each individual) were downloaded and analyzed (GenBank accession number PRJNA241474) (Table 1).

We also filtered out the adapter sequences (> 10 nt aligned to the adapter, allowing ≤ 10% mismatches), low quality reads (i.e. ≥ 10% unidentified nucleotides or > 50% bases having Phred quality < 5) and duplicated reads generated in the library construction process.

Data analysis

Reads mapping

The high quality paired-end reads were mapped to the reference chicken genome (Galgal4.78) using Burrows-Wheeler Aligner (BWA) software (version 0.7.8) [1] with the command 'mem -t 10 -k 32' and BAM alignment files were generated using SAMtools (version 0.1.19) [2].

Next, we improved the alignment results by following steps:

(1) The aligned reads with mismatches ≥ 5 or mapping quality = 0 were removed;

(2) The alignment results were then corrected using Picard (version 1.96) (http://broadinstitute.github.io/picard/) with two core commands. The 'AddOrReplaceReadGroups' command was used to replace all read groups in the INPUT file with a new read group and assign all reads to this group in the OUTPUT BAM. The 'FixMateInformation' command was used to ensure that all mate-pair information was in sync between each read and its mate pair;

(3) Removed potential PCR duplications. If multiple read pairs had identical external coordinates, only the pair with the highest mapping quality was retained;

(4) Realigned reads around the InDels. We downloaded variants registered in chicken dbSNP database (Build 145) from NCBI, and generated a target list of intervals by using the command "RealignerTargetCreator" in package Genome Analysis Toolkit (GATK, version 3.1-1- g07a4bf8) [3]. We further used command "IndelRealigner" to identify regions for realignment where at least one read contains a registered InDel with a cluster of mismatching bases around it.

Consequently, ~21.30 Gb high quality data of each individual mapping to reference chicken genome (Additional file 1: Table S1) were used for subsequent analysis.

SNP calling

We first detected individual SNPs simultaneously confirmed by both SAMtools and GATK. The highly accurate alignment was processed using the 'mpileup' program in SAMtools with the parameters '-C 50 -D -S -m 2 -F 0.002 -d 1000' ('-C 50' is a recommended parameter, '-D' and '-S' are default parameters, '-m 2', '-F 0.002' and '-d 1000' are required paremeters). The variants were then filtered for downstream analysis by requiring a coverage ranging from 4 to

200, a minimum root-mean-square mapping quality of 20 and no gaps present within a 3-bp window. Meanwhile, we detected genomic variants for each bird using GATK with the HaplotypeCaller-based method; before calling variants, the base quality scores were recalibrated using command “BaseRecalibrator”, which provides empirically accurate base quality scores for each base in every read. After SNP calling, we applied hard filter command ‘VariantFiltration’ to exclude potential false-positive variant calls with the parameter ‘--filterExpression "QD < 10.0 || FS > 60.0 || MQ < 40.0 || ReadPosRankSum < -8.0" -G_filter "GQ<20"’. As a result, ~5.26 Mb SNPs for each individual were identified (Additional file 1: Table S2).

Then we merged all individual SNPs into a population SNP-matrix. Finally, we obtained 8.53 Mb highly credible SNPs after using strict criteria with filtering MAF (minor allele frequency) < 0.05 and missing genotype > 10% in chicken population. Subsequently, the package ANNOVAR (version May 20, 2013) [4] was used to annotate SNPs causing nonsense and missense mutations.

Insertions and deletions (InDels) calling

The candidate Indels were called along with SNPs by GATK for 91 individuals. We first sifted structural variations for each sample by GATK with the SelectVariants based method. Then, we applied hard filter command ‘VariantFiltration’ to exclude potential false-positive variant calls with the parameter ‘--filterExpression "QD < 2.0 || FS > 200.0 || ReadPosRankSum < -8.0 || InbreedingCoeff < -0.8"’. Finally, we only retained the 1-5 bp InDels for downsteam analysis. As a result, 323.89-891.64 (K) InDels were identified for each individual, and ANNOVAR was also used to annotate these InDels.

Analysis of the population structure and evolutionary history

Rooted neighbor-joining phylogenetic tree was constructed under the p-distances model in TreeBeST (version 1.9.2) (http://treesoft.sourceforge.net/treebest.shtml), using Japanese quail as an outgroup. The reliability of each branch was evaluated by bootstrapping [5] with 1,000 replicates. The phylogenetic relationships of the individual genomes were also estimated using principle component analysis (PCA) with the population-scale SNPs using the EIGENSOFT (version 5.0) [6] software, and the eigenvectors were obtained from the covariance matrix generated by R function reigen.

Findings

Genetic diversity

We conducted a comparative genomics analysis of 91 chickens from 15 domestic and 2 wild populations (Table 1). The general phenotypic differences between red jungle fowls (RJF), Tibetan fowls and Sichuan local fowls were presented in Additional file 1: Table S3. We identified 3.46-7.52 M SNPs for each population that were confirmed by both SAMtools and

GATK softwares, including 1.10 M novel SNPs in 17 populations that were absent from the current chicken dbSNP database (Figure 1C). Among them, there was 1,812,591 (21.24%) SNPs could be detected in all populations. Allele frequency spectrum for the identified SNPs in each individual was presented in Additional file 1: Table S4. There were 1,398 to 7,977 SNPs specifically detected in a breed/population (Table 1), and a small number of SNPs (ranging from 7 to 79) with a high frequency (>90%) in each breed or population was detected (Additional file 1: Table S5). An average of 601,024 small InDels (1-5bp, ~ 294,493 insertions and 306,551 deletions) for each individual were also identified (Additional file 1: Table S6).

Nucleotide variability (θ_π) and polymorphism (θ_ω) in each population were analyzed using the method of sequence diversity statistics [7]. Compared with Sichuan domestic breeds ($\theta_\pi = 2.35 \times 10^{-3}$ and $\theta_\omega = 2.13 \times 10^{-3}$), Tibetan chicken populations have relatively higher genetic diversity ($\theta_\pi = 2.58 \times 10^{-3}$, $P < 2.2 \times 10^{-16}$ and $\theta_\omega = 2.35 \times 10^{-3}$, $P = 0.656$, Mann-Whitney U test) (Additional file 1: Table S2).

Table 1 Sample information.

Type	Population (n)	Breed	Group[1]	Location, average altitude (m)	Breed/Population specific SNPs
Sichuan local chicken breeds	Pengxian (6)	Pengxian yellow fowl	SW1	Pengxian, Sichuan, 800 m	1,398
	Emei (6)	Emei black fowl	SW1	Leshan, Sichuan, 400 m	2,511
	Jiuyuan (5)	Jiuyuan black fowl	SW1	Yaan, Sichuan, 900 m	1,665
	Muchuan (5)	Muchuan silky fowl	SW1	Muchuan, Sichaun, 500 m	1,654
	Shimian (4)	Shimian caoke fowl	SW1	Shimian, Sichuan, 790 m	1,908
	Tianfu (5)	Tianfu silky fowl	SW1	Chengdu, Sichuan, 540 m	1,651
	Miyi (5)	Miyi fowl	SW2	Panzhihua, Sihucan, 1,400 m	2,123
	Jinyang (6)	Jinyang silky fowl	SW2	Jinyang, Sichuan, 460 m	1,727
Tibetan chicken populations	Aba (5)	Tibetan fowl	TC1 TC2 TC3	Aba, Sichuan, 3,300 m	2,379
	Diqing (6)	Tibetan fowl	TC1 TC2 TC3	Diqing, Yunnan, 3,280 m	2,270
	Ganzi (6)	Tibetan fowl	TC2 TC3	Ganzi, Sichuan, 3,390 m	2,169
	Linzhi (5)	Tibetan fowl	TC1 TC2 TC3	Linzhi, Tibet, 3,100 m	2,499
	Haiyan (6)	Tibetan fowl	TC1 TC2	Haiyan, Qinghai, 3,260 m	2,561
	Shannan (8)	Tibetan fowl	TC1 TC3	Shannan, Tibet, 3,700 m	2,687
Game fowl[2]	Xishuangbanna (8)	Xishuangbanna game fowl	SW3	Xishuangbanna, Yunnan, 1,500 m	4,716
RJF[2]	RJF_Yunnan (4)	Red jungle fowl	RJF	Yunnan	7,977
	RJF_Hainan (1)	Red jungle fowl	RJF	Hainan	2,960

[1] Individual distribution to each group can be found in Additional file 1: Table S1.

[2] The whole-genome sequencing data of eight game fowls and 5 RJFs were downloaded from the NCBI (PRJNA241474).

Population genetics

The neighbor-joining phylogenetic tree revealed the segregation of 15 domestic populations and 2 wild RJF populations into seven distinct clusters (SW1, SW2, SW3, TC1, TC2, TC3 and a RJF group) (Figure 1D,E). A similar pattern of clustering was also observed based on principal component analysis (PCA) using EIGENSOFT package [6]. Different from a previous report on the two independent origins of Tibetan chickens [8], we revealed the presence of at least three distinct clusters among the six geographically representative

populations of Tibetan fowls: the fowls inhabiting Tibet and Qinghai (cluster TC1) were genetically closer to RJF, while the TCs inhabiting Yunnan and Sichuan (cluster TC2 and TC3) were closer to the domestic populations (Figure 1D). These distinct distribution patterns and expansion signatures suggested that the divergent Tibetan clades may have originated from different regions, such as Yunnan, southwest China and/or surrounding areas [8]. We found that TC2 and TC3 clustered with other Sichuan local chicken breeds, which may be attributable to shared ancestral polymorphism and/or recent introgression events by way of possible crossbreeding between TC with the geographically neighboring Sichuan local chickens. Although this inference is consistent with recent breeding activities in Tibet plateau [8], further analysis are required to explore the introgression between them.

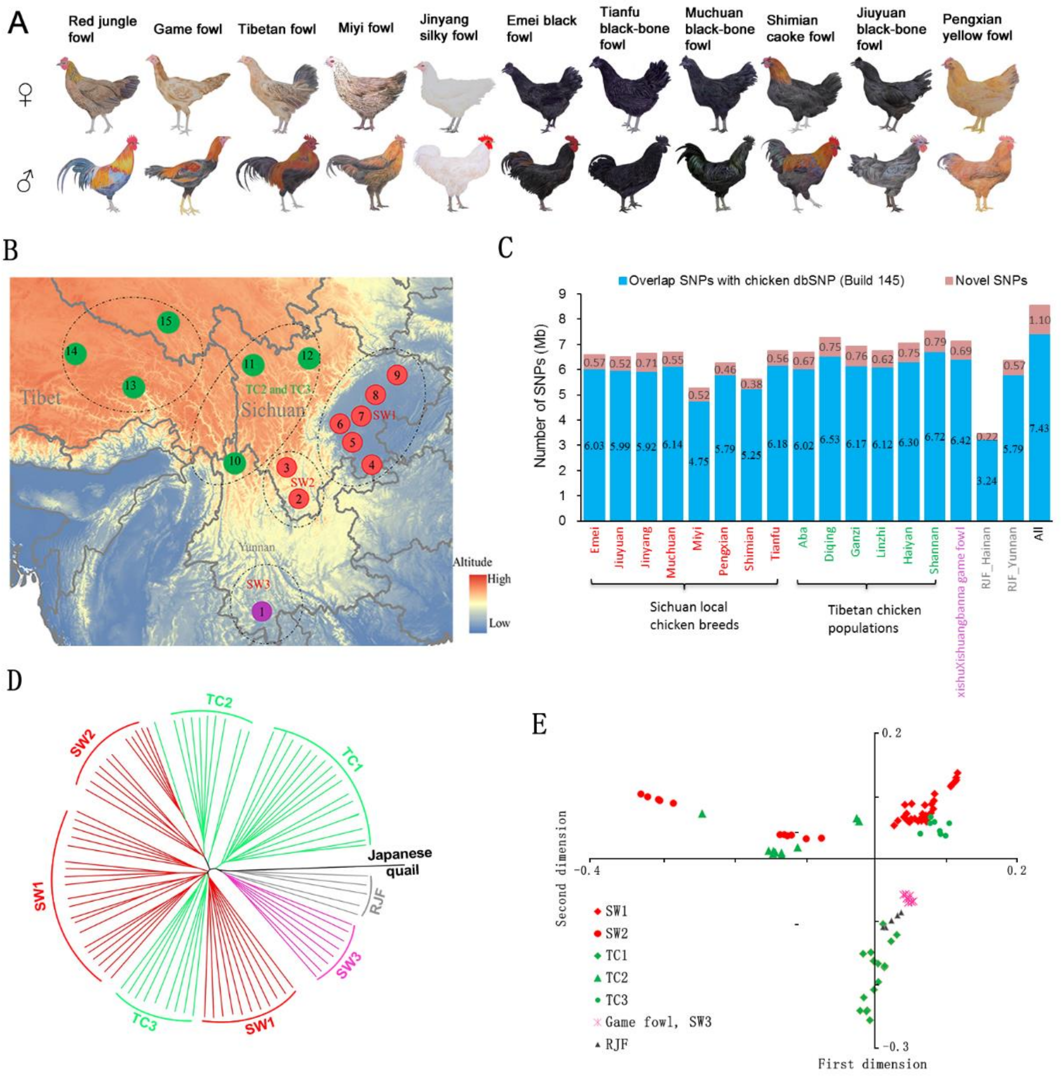

Figure 1 Population genetics of studied chickens.

(A**)** The striking phenotypic differences among the RJF and 10 domestic chicken breeds analyzed in this study. (B) Geographic distribution of the chicken populations. Red and green localities represent eight lowland and six highland chicken populations sampled in this study, respectively. 1-15 represent Xishuangbanna, Miyi, Jinyang, Muchuan, Emei, Shimian, Jiuyuan, Tianfu, Pengxian, Diqing, Ganzi, Aba, Shannan, Linzhi and Haiyan, respectively. (C) Comparison of identified SNPs in the 15 domestic chicken populations and red jungle fowls with the public database of chicken variants (dbSNP, Build 145). (D) Rooted neighbor-joining phylogenetic tree with the neighbor-joining method, using Japanese quail as an outgroup. The reliability of each branch

was evaluated by bootstrapping with 1,000 replicates. Different groups of chicken populations: Sichuan domestic chickens (red), Tibetan chickens (green), the Xishuangbanna game fowls (purple), RJFs (grey) and Japanese quail (black). (E) Principal component plots. The first dimension and second dimension are shown. The fraction of the variance explained was 8.91% for eigenvector 1 ($P<0.05$, Tracy-Widom test) and 7.43% for eigenvector 2 ($P<0.05$, Tracy-Widom test).

Conclusion

Understanding the nature of diversifying selection, especially detecting selection signatures, and identifying genes in a genome that are, or have been, under selection have been the hot topics of interests. This study provides comparative genomic landscape of variations in 17 chicken populations to understand genetic variations underlying the phenotypic diversity of chicken breeds/populations. This data will serve as a valuable resource for investigating diversifying selection and candidate genes for selective breeding in chicken.

Availability of supporting data

The sequencing data for this project have been deposited in the NCBI sequence read archive (SRA) under accession number SRP067615. All supplementary figures and tables are provided in Additional file 1.

Additional file

Additional file 1: Table S1, Table S2, Table S3, Table S4, Table S5 and Table S6. (xlsx 741KB). **Table S1.** A summary of the chickens used in this study: regions of collection/popularization and coverage and mean depth of resequencing. **Table S2.** SNPs annotation and genetic diversity of 17 chicken populations analyzed in this study. **Table S3.** The general phenotypic differences between RJF, Tibetan and Sichuan local chickens. **Table S4.** Allele frequency spectrum for the identified SNPs in each individual. **Table S5.** The frequency distribution of breed/population specific SNPs. **Table S6.** The small indels (1-5bp) in 91 individuals.

Funding

This work was supported by China Agricultural Research System (CARS-41), the 12th Five Year Plan for breeding program in Sichuan-Selective breeding of new breeds and the synthetic strains in laying hens (2011NZ0099-7), National Natural Science Foundation of China (31402063), Sichuan Provincial Department of Science and Technology Program (2015JQO023) and the National Program for Support of Top-notch Young Professionals and the Young Scholars of the Yangtze River.

Authors' contributions

Q.Z., and MZ.L. designed and supervised the project. B.C., M.L., H.Y., Y.W., X.Z., G.Z., U.G., MJ.L., L.Z., M.Y., R.J., R.L., and X.Z collected and generated the data, and performed the preliminary bioinformatic analyses. T.C., S.T., Z.X., L.J., Q.T., H.X., and X.Z. filtered the data and performed the majority of the population genetic analysis. D.L. and T.C. wrote the

manuscript.

Competing financial interests

The authors declare no competing financial interests.

References

1. Li H,Durbin R. Fast and accurate long-read alignment with Burrows–Wheeler transform. Bioinformatics. 2010; 26: 589-595.
2. Li H, Handsaker B, Wysoker A, Fennell T, Ruan J, Homer N, et al. The sequence alignment/map format and SAMtools. Bioinformatics. 2009; 25: 2078-2079.
3. McKenna A, Hanna M, Banks E, Sivachenko A, Cibulskis K, Kernytsky A, et al. The Genome Analysis Toolkit: a MapReduce framework for analyzing next-generation DNA sequencing data. Genome Res. 2010; 20: 1297-1303.
4. Conesa A, Götz S, García-Gómez JM, Terol J, Talón M,Robles M. Blast2GO: a universal tool for annotation, visualization and analysis in functional genomics research. Bioinformatics. 2005; 21: 3674-3676.
5. Felsenstein J. Confidence limits on phylogenies: an approach using the bootstrap. Evolution. 1985783-791.
6. Nick P, Price AL,David R. Population structure and eigenanalysis. Plos Genetics. 2006; 2: 2074--2093.
7. Nei M, .,Li WH. Mathematical model for studying genetic variation in terms of restriction endonucleases. Proc Natl Acad Sci U S A. 1979; 76: 5269-5273.
8. Ming-Shan W, Yan L, Min-Sheng P, Li Z, Zong-Ji W, Qi-Ye L, et al. Genomic Analyses Reveal Potential Independent Adaptation to High Altitude in Tibetan Chickens. Molecular Biology & Evolution. 2015; 32: 1880-9.

Figure

Click here to download Figure giga Figure 1.png

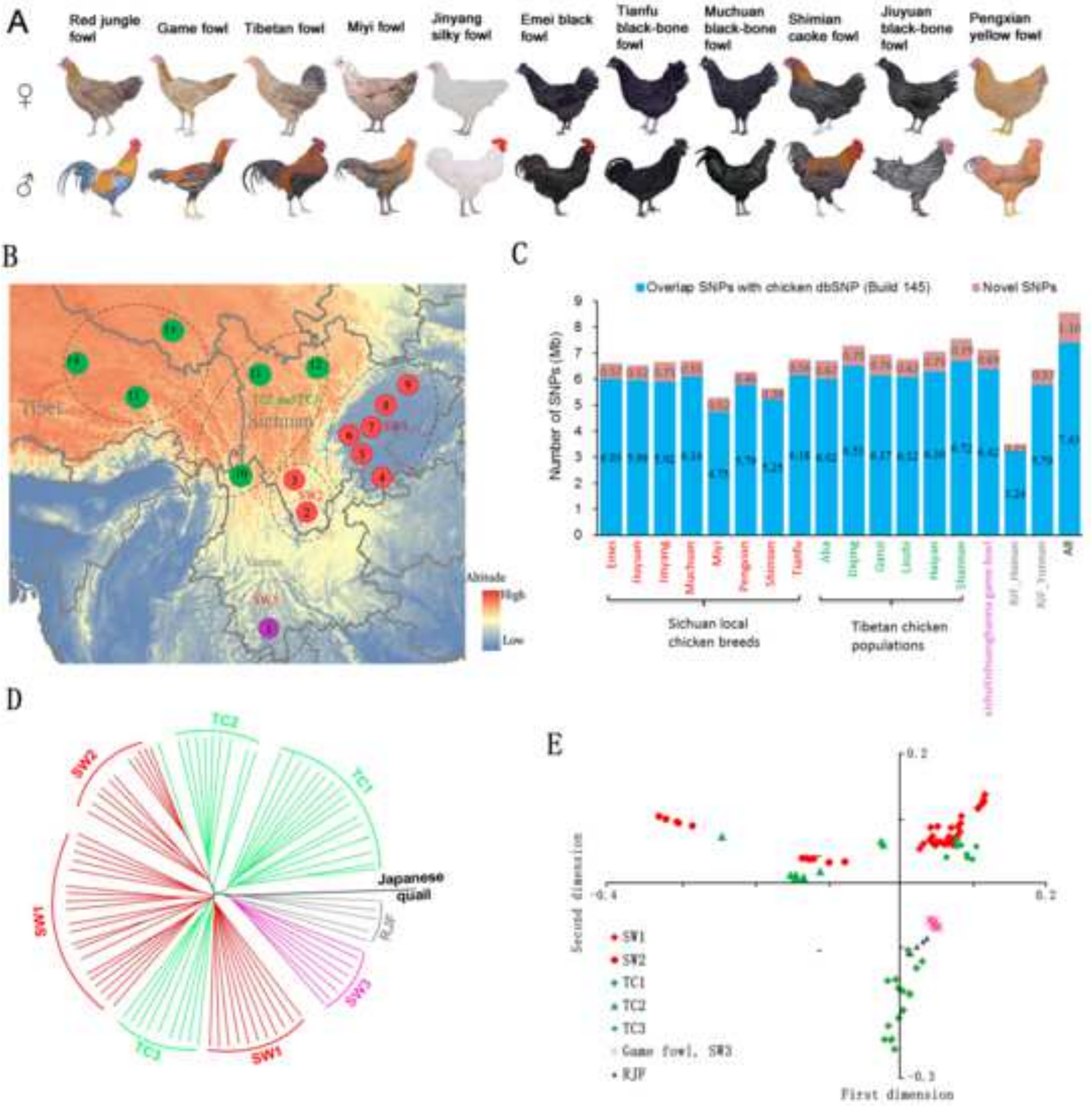

Click here to access/download
Supplementary Material
Aditional file 1.xlsx

Personal Cover Click here to download Personal Cover coverletter.docx

Sichuan Agricultural University

Institute of Animal Genetics and Breeding Sciences

Chengdu 611130, P. R. China
Tel: (0086) 028-86290991, E-mail: zhuqingsicau@163.com

Editor
GigaScience

October 25, 2016

Dear Editor-in-Chief，

I am pleased to submit our manuscript entitled “**Genomic data for 78 chickens from 14 populations**” for your consideration for publication in GigaScience.

Population genomic variations through diversifying selection have not been reported, although chicken genome has been sequenced. In this study, we resequenced the whole genomes from a 78 domestic chickens including 42 lowland chickens from 8 phenotypically diverse breeds and 36 Tibetan chickens from 6 major highland locations in Qinghai-Tibet Plateau, and present provide a population-level genome landscape of genetic variations in chickens. This data can be further used to provide new insights into diversifying selection and candidate genes for selective breeding. I am sure this is good topic for your journal. I am pleased to submit it for your consideration for publication in your journal.

The manuscript has not been submitted or is under consideration for publication elsewhere now. All authors agree to submit to your journal.

I look forward to hearing from you. Thank you for your consideration!

With best regards,

Qingzhu.
