# Supplementary material for: Genomic data for 78 chickens from 14 populations
Source: Gigascience. 2017 Apr 18;6(6):1–5. doi: 10.1093/gigascience/gix026 (PMC5449643; doi:10.1093/gigascience/gix026)
Supplement: GIGA-D-16-00092_Revision_2.pdf [file gix026_GIGA-D-16-00092_Revision_2.pdf]

Manuscript
Click here to download Manuscript MS-revised-R2.docx

Genomic data for 78 chickens from 14 populations

Diyan Li[1†], Tiandong Che[1†], Binlong Chen[1†], Shilin Tian[1,2†], Xuming Zhou[3†], Guolong Zhang[4†], Miao Li[1], Uma Gaur[1], Yan Li[1], Majing Luo[5], Long Zhang[1], Zhongxian Xu[1], Xiaoling Zhao[1], Huadong Yin[1], Yan Wang[1], Long Jin[1], Qianzi Tang[1], Huailiang Xu[1], Mingyao Yang[1], Rongjia Zhou[5], Ruiqiang Li[2], Qing Zhu[1] and Mingzhou Li[1]

[1] Institute of Animal Genetics and Breeding, College of Animal Science and Technology, Sichuan Agricultural University, Chengdu, China

[2] Novogene Bioinformatics Institute, Beijing, China

[3] Division of Genetics, Department of Medicine, Brigham and Women's Hospital, Harvard Medical School, Boston, USA

[4] Department of Animal Science, Oklahoma State University, Stillwater, Oklahoma, USA

[5] Hubei Key Laboratory of Cell Homeostasis, Laboratory of Molecular and Developmental Genetics, College of Life Sciences, Wuhan University, Wuhan, China

[†] These authors contributed equally to this work.

Correspondence: zhuqingsicau@163.com; mingzhou.li@sicau.edu.cn.

Abstract

Background: Since the domestication of the red jungle fowls (*Gallus gallus*) (dating back to ~10,000 B.P.) in Asia, domestic chickens (*Gallus gallus domesticus*) have been subjected to the combined effects of natural selection and human-driven artificial selection; this has resulted in marked phenotypic diversity in a number of traits, including behavior, body composition, egg production and skin color. Population genomic variations through diversifying selection have not been fully investigated.

Findings: The whole genomes of 78 domestic chickens were sequenced to an average of 18-fold coverage for each bird. By combining this data with publicly available genomes of 5 wild red jungle fowls and 8 Xishuangbanna game fowls, we conducted a comprehensive comparative genomics analysis of 91 chickens from 17 populations. After aligning ~21.30 gigabases (Gb) of high quality data from each individual to the reference chicken genome, we identified ~6.44 million (M) SNPs for each population. These SNPs included 1.10 M novel SNPs in 17 populations that were absent in the current chicken dbSNP (Build 145) entries.

Conclusions: The current data is important for population genetics and further studies in chicken, and will serve as a valuable resource for investigating diversifying selection and candidate genes for selective breeding in chicken.

Keywords: Chicken, Genetic diversity, Population genomics, Whole-genome resequencing

Data description

Genome sequencing and sequence filtering

The 78 blood samples (36 Tibetan fowls from the Qinghai-Tibet Plateau and 42 domestic fowls from Szechwan Basin) (Figure 1) were collected from the wing vein. The animal handling experiments were approved by the Institutional Animal Care and Use Committee of Sichuan Agricultural University under permit number YCS-B20100804. Genomic DNA was extracted from these samples following standard procedures. In total, we generated ~1.69 trillion bases of resequencing data of the whole genomes from 78 birds (18.03-fold coverage for each

individual) on the Illumina Hiseq 2500 platform (Additional file 1:Table S1). In addition, previously published genome sequence data from 5 red jungle fowls (RJF) and 8 Xishuangbanna game fowls (~16.6-fold coverage for each individual) were downloaded and analyzed (GenBank accession number PRJNA241474) (Figure 1).

We also filtered out the adapter sequences (> 10 nt aligned to the adapter, allowing ≤ 10% mismatches), low quality reads (i.e. ≥ 10% unidentified nucleotides or > 50% bases having Phred quality < 5) and duplicated reads generated in the library construction process.

Data analysis

Reads mapping

The high quality paired-end reads were mapped to the reference chicken genome (Galgal4.78) using Burrows-Wheeler Aligner (BWA) software (version 0.7.8) [1] with the command 'mem -t 10 -k 32' and BAM alignment files were generated using SAMtools (version 0.1.19) [2].

Next, we improved the alignment results by the following steps:

(1) The aligned reads with mismatches ≥ 5 or mapping quality = 0 were removed;

(2) The alignment results were then corrected using Picard (version 1.96) (http://broadinstitute.github.io/picard/) with two core commands. The 'AddOrReplaceReadGroups' command was used to replace all read groups in the INPUT file with a new read group and assign all reads to this group in the OUTPUT BAM. The 'FixMateInformation' command was used to ensure that all mate-pair information was in sync between each read and its mate pair;

(3) Removed potential PCR duplications. If multiple read pairs had identical external coordinates, only the pair with the highest mapping quality was retained;

(4) Realigned reads around the InDels. We downloaded variants registered in chicken dbSNP database (Build 145) from NCBI, and generated a target list of intervals by using the command "RealignerTargetCreator" in package Genome Analysis Toolkit (GATK, version 3.1-1- g07a4bf8) [3]. We further used the command "IndelRealigner" to identify regions for realignment where at least one read contains a registered InDel with a cluster of mismatching bases around it.

Consequently, ~21.30 Gb high quality data of each individual mapping to reference chicken genome (Additional file 1: Table S1) were used for subsequent analysis.

SNP calling

We first detected individual SNPs simultaneously confirmed by both SAMtools and GATK. The highly accurate alignment was processed using the 'mpileup' program in SAMtools with the parameters '-C 50 -D -S -m 2 -F 0.002 -d 1000' ('-C 50' is a recommended parameter, '-D' and '-S' are default parameters, '-m 2', '-F 0.002' and '-d 1000' are required paremeters). The

variants were then filtered for downstream analysis by requiring a coverage ranging from 4 to 200, a minimum root-mean-square mapping quality of 20 and no gaps present within a 3-bp window. Meanwhile, we detected genomic variants for each bird using GATK with the HaplotypeCaller-based method; before calling variants, the base quality scores were recalibrated using command “BaseRecalibrator”, which provides empirically accurate base quality scores for each base in every read. After SNP calling, we applied hard filter command ‘VariantFiltration’ to exclude potential false-positive variant calls with the parameter ‘--filterExpression "QD < 10.0 || FS > 60.0 || MQ < 40.0 || ReadPosRankSum < -8.0" -G_filter "GQ<20"’. As a result, ~6.44 Mb SNPs for each breed/population were identified (Additional file 1: Table S2).

Then we merged all individual SNPs into a population SNP-matrix. Finally, we obtained 8.53 Mb highly credible SNPs after using strict criteria with filtering MAF (minor allele frequency) < 0.05 and missing genotype > 10% in chicken population. Subsequently, the package ANNOVAR (version May 20, 2013) [4] was used to annotate SNPs causing nonsense and missense mutations.

Insertions and deletions (InDels) calling

The candidate InDels were called along with SNPs by GATK for 91 individuals. We first sifted structural variations for each sample by GATK with the SelectVariants based method. Then, we applied hard filter command ‘VariantFiltration’ to exclude potential false-positive variant calls with the parameter ‘--filterExpression "QD < 2.0 || FS > 200.0 || ReadPosRankSum < -8.0 || InbreedingCoeff < -0.8"’. Finally, we only retained the 1-30 bp InDels for downsteam analysis.

Analysis of the population structure and evolutionary history

Rooted neighbor-joining phylogenetic tree was constructed under the p-distances model in TreeBeST (version 1.9.2) (http://treesoft.sourceforge.net/treebest.shtml), using Japanese quail as an outgroup. The reliability of each branch was evaluated by bootstrapping [5] with 1,000 replicates. The phylogenetic relationships of the individual genomes were also estimated using principle component analysis (PCA) with the population-scale SNPs using the EIGENSOFT (version 5.0) [6] software, and the eigenvectors were obtained from the covariance matrix generated by R function reigen.

Findings

Genetic diversity

A total of 7.43 Mb of SNPs out of 8.53 Mb highly credible SNPs were already present in chicken dbSNP database (overlapped SNPs) and 1.10 Mb SNPs were assigned as novel ones. All 1.10 Mb novel SNPs have been submitted to dbSNP (accession numbers from ss2585830405 to ss2586846514 and ss2137077162; see Additional file 2). We further

conducted a comparative genomics analysis of 91 chickens from 15 domestic and 2 wild populations (Figure 1). The general phenotypic differences between red jungle fowls (RJF), Tibetan fowls and Sichuan local fowls are shown in Additional file 1: Table S3. We identified 3.46-7.52 Mb SNPs for each breed/population that were confirmed by both SAMtools and GATK softwares (Additional file 1: Table S2). There were 1,398 to 7,977 SNPs specifically detected in a breed/population (Figure 1). Nucleotide variability (θ_π) and polymorphism (θ_ω) in each population were analyzed using the method of sequence diversity statistics [7]. Compared with Sichuan local chicken breeds (θ_π = 2.35 $\times 10^{-3}$ and θ_ω = 2.13 $\times 10^{-3}$), Tibetan chicken populations have relatively higher genetic diversity (θ_π = 2.58 $\times 10^{-3}$, $P < 2.2 \times 10^{-16}$ and θ_ω = 2.35 $\times 10^{-3}$, P = 0.656, Mann-Whitney U test) (Additional file 1: Figure S1).

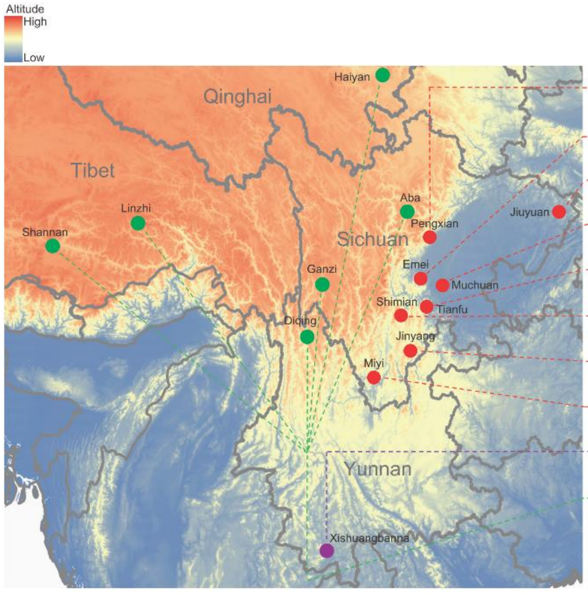

Altitude	♂	♀	Breed	Population (n)	Group[1]	Overlapped SNPs(Mb)	Novel SNPs(Mb)	Breed/Population specific SNPs	
<1km			Pengxian yellow fowl	Pengxian (6)	Cluster3	5.79	0.46	1,398	Sichuan local chicken breeds
<1km			Emei black fowl	Emei (6)	Cluster3	6.03	0.57	2,511	
<1km			Jiuyuan black-bone fowl	Jiuyuan (5)	Cluster3	5.99	0.52	1,665	
<1km			Muchuan black-bone fowl	Muchuan (5)	Cluster3	6.14	0.55	1,654	
<1km			Tianfu black-bone fowl	Tianfu (5)	Cluster3	6.18	0.56	1,651	
<1km			Shimian caoke fowl	Shimian (4)	Cluster3	5.25	0.38	1,908	
<1km			Jinyang silky fowl	Jinyang (6)	Cluster2	5.92	0.71	1,727	
1-2km			Miyi fowl	Miyi (5)	Cluster2	4.75	0.52	2,123	
1-2km			Xishuangbanna game fowl[2]	Xishuangbanna (8)	Cluster1	6.42	0.69	4,716	
3-4km			Tibetan fowl	Aba (5)	Cluster1,2,3	6.02	0.67	2,379	Tibetan chicken populations
			Tibetan fowl	Diqing (6)	Cluster1,2,3	6.53	0.75	2,270	
			Tibetan fowl	Ganzi (6)	Cluster2,3	6.17	0.76	2,169	
			Tibetan fowl	Linzhi (5)	Cluster1,2,3	6.12	0.62	2,499	
			Tibetan fowl	Haiyan (6)	Cluster1,2	6.30	0.75	2,561	
			Tibetan fowl	Shannan (8)	Cluster1,3	6.72	0.79	2,687	
			Red jungle fowl[2]	RJF_Yunnan (4)	Cluster1	5.79	0.57	7,977	
			Red jungle fowl[2]	RJF_Hainan (1)	Cluster1	3.24	0.22	2,960	

Figure 1. Sample information and comparison of identified SNPs in each breed/population with the chicken variants database (dbSNP, Build 145). Overlapped SNPs are SNPs already in chicken dbSNP. The map displayed here is the geographic distribution of domestic chicken populations, numbers above the dashed lines are altitudes. Red and green localities represent eight lowland and six highland chicken populations respectively, sampled in this study. [1] Individual distribution to each group can be found in Additional file 1: Table S1. [2] The whole-genome sequencing data of eight game fowls and 5 RJFs were downloaded from the NCBI.

As shown in Additional file 1: Figure S2, although most novel SNPs (89.02%) had a low allele frequency (<0.2 of 91 individuals) compared with the overlapped SNPs (44.02%), only 9,918 (0.88% of 1.10 M) novel SNPs were specifically detected in one breed/population (at least in an individual). These novel SNPs also exhibited a comparable sequencing depth with the overlapped SNPs (median of normalized depth of 1.14 versus 1.06) (Additional file 1:

Figure S3). In addition, we observed more than 75% of the novel SNPs and 86% of the overlapped SNPs were in non-repeat regions. These results suggest the novel SNPs will serve as a potentially valuable resource for further chicken studies.

Overall distribution of the lengths of insertions and deletions (InDels) showed that more than 80% of the InDels were 1-5 bp in length (Additional file 1: Figure S4). Repetitive elements (10.61% of the genome and containing ~15.70% of InDels) are an important source of structural variation in chicken genome (Additional file 1: Figure S5). About a half of InDels (48.39% to 51.52%) were occurred in the intergenic regions (588.65 Mb and 56.23% of the genome). The introns (403.35 Mb and containing ~43.86% of InDels) showed higher incidence of InDels than the coding sequences (25.81 Mb and containing ~1.77% of InDels) (Additional file 1: Figure S6). We observed an enrichment of short InDels (1-15 bp in length) in coding sequences that were multiples of 3 bp compared to whole genome sequences, which is expected to preserve the reading frame (Additional file 1: Figure S7).

Population genetics

The neighbor-joining phylogenetic tree revealed the segregation of 15 domestic populations and 2 wild RJF populations into three distinct clusters (cluster 1, cluster 2 and cluster 3) (Figure 2A). A similar pattern of clustering (Figure 2B) was also observed based on principal component analysis (PCA) using EIGENSOFT package [6]. Different from a previous report on the two independent origins of Tibetan chickens [8], we revealed the presence of at least three distinct clusters among the six geographically representative populations of Tibetan fowls: the fowls inhabiting Tibet and Qinghai (in cluster 1) were genetically closer to RJF, while the Tibetan chickens inhabiting Yunnan and Sichuan (cluster 2 and 3) were closer to the domestic populations (Figure 1). These distinct distribution patterns and expansion signatures suggested that the divergent Tibetan clades may have originated from different regions, such as Yunnan, southwest China and/or surrounding areas [8]. We found that many Tibetan chickens clustered with other Sichuan local chicken breeds in cluster 2 and cluster 3, which may be attributable to shared ancestral polymorphism and/or recent introgression events by way of possible crossbreeding between Tibetan chicken with the geographically neighboring Sichuan local chickens. Although this inference is consistent with recent breeding activities in Tibet plateau [8], further analysis are required to explore the introgression between them.

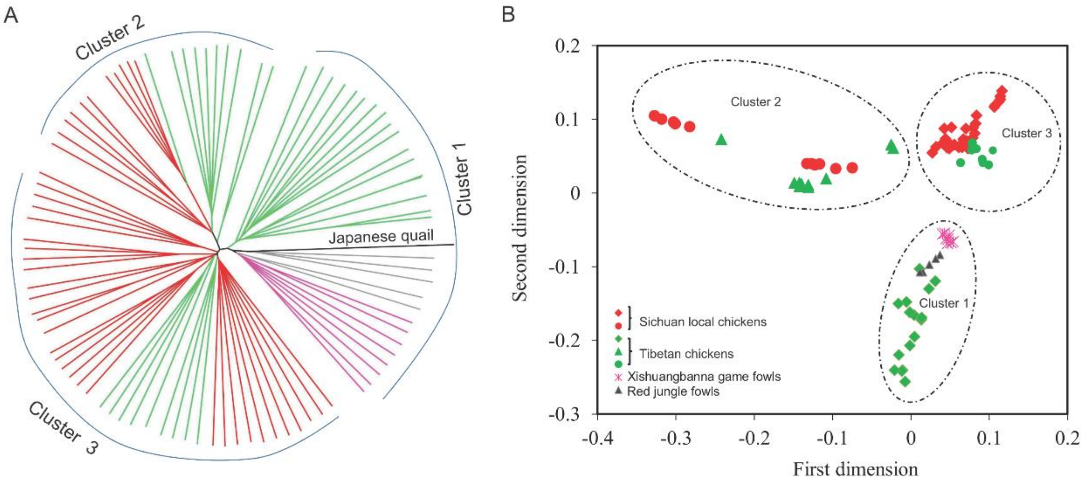

Figure 2. Population genetics of studied chickens. (A) Rooted neighbor-joining phylogenetic

tree with the neighbor-joining method, using Japanese quail as an outgroup. The reliability of each branch was evaluated by bootstrapping with 1,000 replicates. Different groups of chicken populations: Sichuan local chickens (red), Tibetan chickens (green), the Xishuangbanna game fowls (purple), RJFs (grey) and Japanese quail (black). (B) Principal component plots. The first dimension and second dimension are shown. The fraction of the variance explained was 8.91% for eigenvector 1 (P<0.05, Tracy-Widom test) and 7.43% for eigenvector 2 (P<0.05, Tracy-Widom test).

Conclusion

Understanding the nature of diversifying selection, especially detecting selection signatures, and identifying genes in a genome that are, or have been, under selection have been the hot topics of interests. This study provides comparative genomic landscape of variations in 17 chicken populations to understand genetic variations underlying the phenotypic diversity of chicken breeds/populations. This data will serve as a valuable resource for investigating diversifying selection and candidate genes for selective breeding in chicken.

Availability of supporting data

The sequencing data for this project have been deposited in the NCBI sequence read archive (SRA) under accession number SRP067615. All supplementary Figures and Tables are provided in Additional file 1.

Additional file

Additional file 1: Table S1, Table S2, Table S3, Figure S1, Figure S2, Figure S3, Figure S4 and Figure S5. (doc 1.3 MB). **Table S1.** A summary of the chickens used in this study: regions of collection/popularization and coverage and mean depth of resequencing. **Table S2.** SNPs annotation and genetic diversity of 17 chicken populations analyzed in this study. **Table S3.** The general phenotypic differences between red jungle fowls, Tibetan and Sichuan local chickens. **Figure S1.** Average nucleotide polymorphism (θw) and nucleotide diversity ($\theta \pi$) among Sichuan local chickens, Tibetan chickens and red jungle fowls. **Figure S2.** Allele frequency spectra in 91 birds and Number of alleles distribute in 1 to 17 chicken breeds/populations. **Figure S3.** Comparison of sequencing depth between the SNPs that are already in dbSNP (overlapped SNPs) and novel SNPs. **Figure S4.** Overall distribution of the lengths of InDels (1-30 bp). **Figure S5.** Percentage composition of InDels in repeat elements. **Figure S6.** Percentage distribution (A) and probability (B) for InDels across different genomic elements. **Figure S7.** Length distribution of small InDels in the whole genome (A) and coding sequence (CDS) regions (B).

Additional file 2: Accession numbers of 1.10 Mb novel SNPs. (txt 20.3 Mb)

Funding

This work was supported by China Agricultural Research System (CARS-41), the 12th Five Year Plan for breeding program in Sichuan-Selective breeding of new breeds and the synthetic strains in laying hens (2011NZ0099-7), National Natural Science Foundation of China

(31402063), Sichuan Provincial Department of Science and Technology Program (2015JQO023) and the National Program for Support of Top-notch Young Professionals and the Young Scholars of the Yangtze River.

Authors' contributions

Q.Z., and MZ.L. designed and supervised the project. B.C., M.L., H.Y., Y.W., X.Z., G.Z., U.G., MJ.L., L.Z., M.Y., R.J., R.L., and X.Z collected and generated the data, and performed the preliminary bioinformatic analyses. T.C., S.T., Y.L., Z.X., L.J., Q.T., H.X., and X.Z. filtered the data and performed the majority of the population genetic analysis. D.L. and T.C. wrote the manuscript.

Competing financial interests

The authors declare no competing financial interests.

References

1. Li H,Durbin R. Fast and accurate long-read alignment with Burrows–Wheeler transform. Bioinformatics. 2010; 26: 589-595.
2. Li H, Handsaker B, Wysoker A, Fennell T, Ruan J, Homer N, et al. The sequence alignment/map format and SAMtools. Bioinformatics. 2009; 25: 2078-2079.
3. McKenna A, Hanna M, Banks E, Sivachenko A, Cibulskis K, Kernytsky A, et al. The Genome Analysis Toolkit: a MapReduce framework for analyzing next-generation DNA sequencing data. Genome Res. 2010; 20: 1297-1303.
4. Conesa A, Götz S, García-Gómez JM, Terol J, Talón M,Robles M. Blast2GO: a universal tool for annotation, visualization and analysis in functional genomics research. Bioinformatics. 2005; 21: 3674-3676.
5. Felsenstein J. Confidence limits on phylogenies: an approach using the bootstrap. Evolution. 1985783-791.
6. Nick P, Price AL,David R. Population structure and eigenanalysis. Plos Genetics. 2006; 2: 2074--2093.
7. Nei M, .,Li WH. Mathematical model for studying genetic variation in terms of restriction endonucleases. Proc Natl Acad Sci U S A. 1979; 76: 5269-5273.
8. Ming-Shan W, Yan L, Min-Sheng P, Li Z, Zong-Ji W, Qi-Ye L, et al. Genomic Analyses Reveal Potential Independent Adaptation to High Altitude in Tibetan Chickens. Molecular Biology & Evolution. 2015; 32: 1880-9.

Figure 1

Click here to download Figure Figure 1.pdf

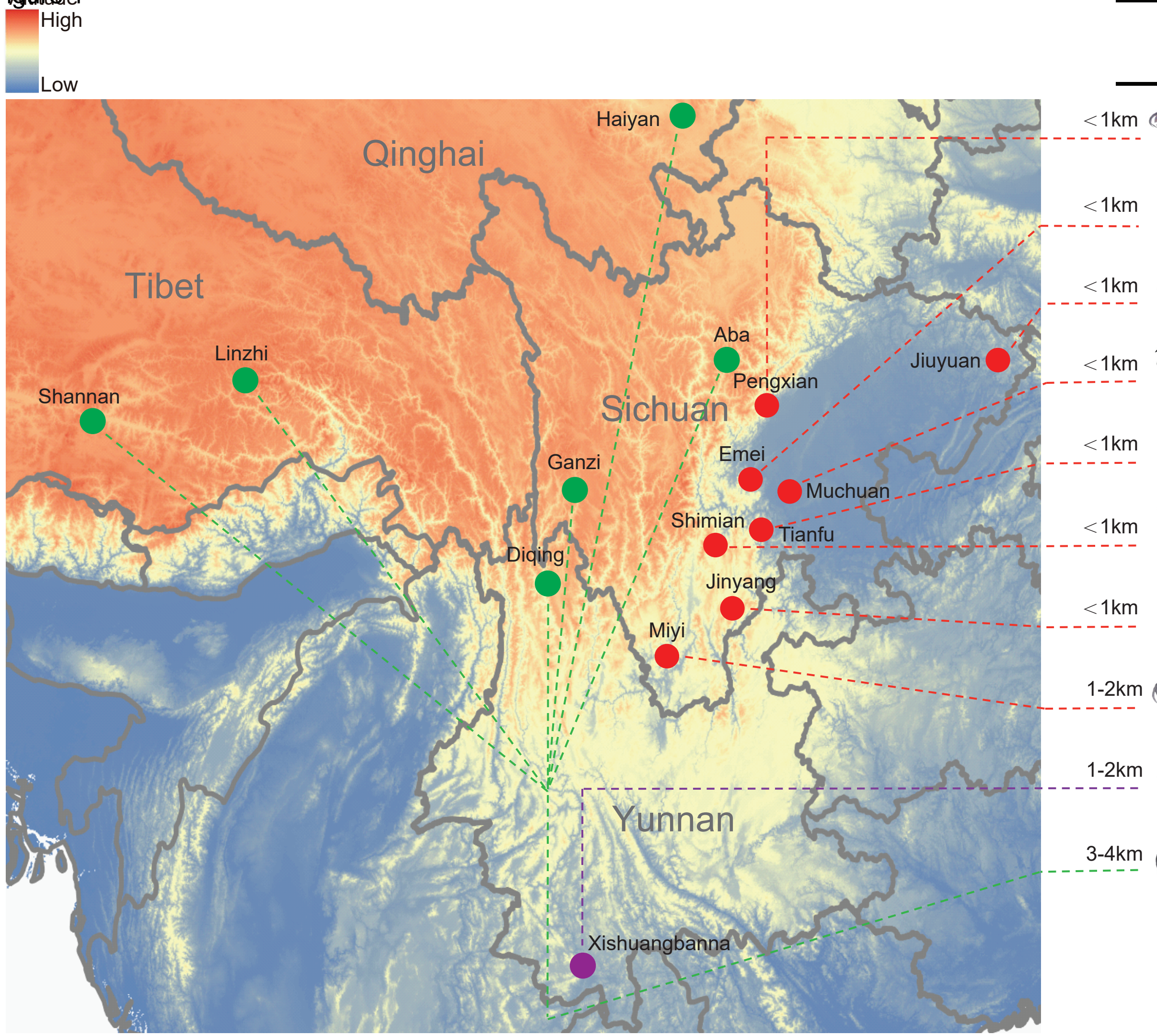

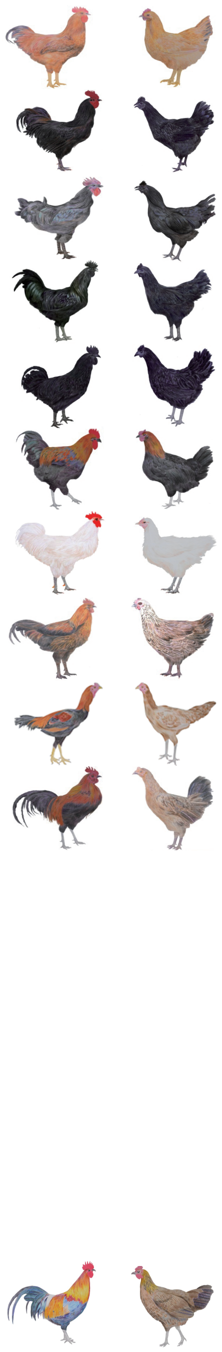

♂	♀	Breed	Population (n)	Group[1]	Overlapped SNPs(Mb)	Novel SNPs(Mb)	Breed/Population specific SNPs	
		Pengxian yellow fowl	Pengxian (6)	Cluster3	5.79	0.46	1,398	Sichuan local chicken breeds
		Emei black fowl	Emei (6)	Cluster3	6.03	0.57	2,511	
		Jiuyuan black-bone fowl	Jiuyuan (5)	Cluster3	5.99	0.52	1,665	
		Muchuan black-bone fowl	Muchuan (5)	Cluster3	6.14	0.55	1,654	
		Tianfu black-bone fowl	Tianfu (5)	Cluster3	6.18	0.56	1,651	
		Shimian caoke fowl	Shimian (4)	Cluster3	5.25	0.38	1,908	
		Jinyang silky fowl	Jinyang (6)	Cluster2	5.92	0.71	1,727	
		Miyi fowl	Miyi (5)	Cluster2	4.75	0.52	2,123	
		Xishuangbanna game fowl[2]	Xishuangbanna (8)	Cluster1	6.42	0.69	4,716	
		Tibetan fowl	Aba (5)	Cluster1,2,3	6.02	0.67	2,379	Tibetan chicken populations
		Tibetan fowl	Diqing (6)	Cluster1,2,3	6.53	0.75	2,270	
		Tibetan fowl	Ganzi (6)	Cluster2,3	6.17	0.76	2,169	
		Tibetan fowl	Linzhi (5)	Cluster1,2,3	6.12	0.62	2,499	
		Tibetan fowl	Haiyan (6)	Cluster1,2	6.30	0.75	2,561	
		Tibetan fowl	Shannan (8)	Cluster1,3	6.72	0.79	2,687	
		Red jungle fowl[2]	RJF_Yunnan (4)	Cluster1	5.79	0.57	7,977	
		Red jungle fowl[2]	RJF_Hainan (1)	Cluster1	3.24	0.22	2,960	

Figure 2

Click here to download Figure Figure 2 .pdf

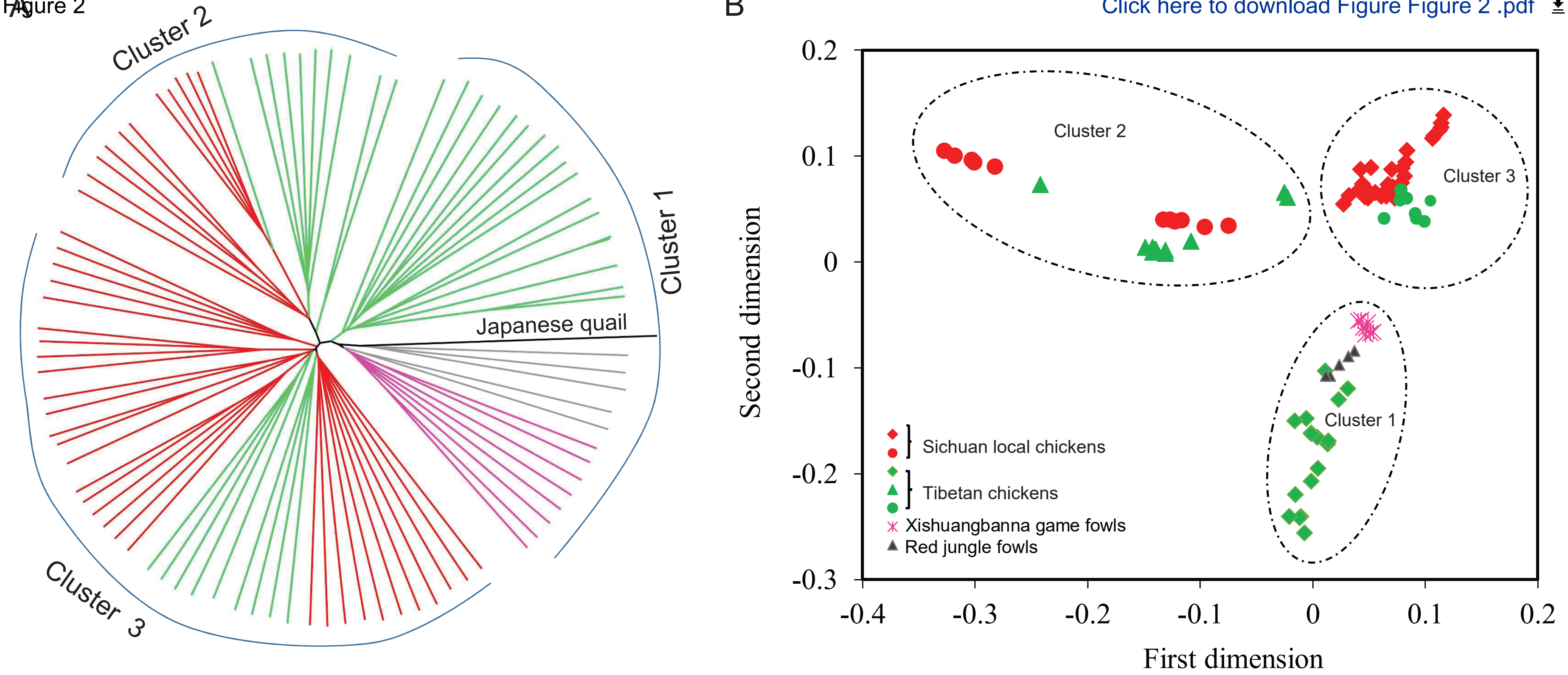

Click here to access/download
Supplementary Material
Aditional file 1-R2.docx

Click here to access/download
Supplementary Material
Additional file 2.txt
