# Supplementary material for: Genomic data for 78 chickens from 14 populations
Source: Gigascience. 2017 Apr 18;6(6):1–5. doi: 10.1093/gigascience/gix026 (PMC5449643; doi:10.1093/gigascience/gix026)
Supplement: Supplemental material — The sequencing data for this project have been deposited in the NCBI sequence read archive (SRA) under accession number SRP067615. Additional data, including sequence variations in variant call format, are available in the GigaScience repository, GigaDB [9]. All supplementary figures and tables are provided in Additional File 1: Table S1. A summary of the chickens used in this study: regions of collection/population and sequencing depths. Table S2. SNP annotation and genetic diversity of the 17 chicken populations analyzed in this study. Table S3. The general phenotypic differences between red jungle fowls, Tibetan chickens, and Sichuan local chickens. Figure S1. Average nucleotide polymorphism (θw) and nucleotide diversity (θπ) among Sichuan local chickens, Tibetan chickens, and red jungle fowls. Figure S2. Allele frequency spectra in 91 birds and number of alleles distribute in 1 to 17 chicken breeds/populations. Figure S3. Comparison of read depth between the known and novel SNPs. Figure S4. Overall distribution of the lengths of InDels (1–30 bp). Figure S5. Percentage composition of InDels in repeat elements. Figure S6. Percentage distribution (A) and probability (B) for InDels across different genomic elements. Figure S7. Length distribution of small InDels in the whole genome (A) and coding sequence (CDS) regions (B). Additional File 2 includes Accession numbers of 1.10 Mb novel SNPs (txt 20.3 Mb). [file gix026_Supp.zip › Aditional file 1-R3.docx]

**Genomic data for 78 chickens from 14 populations**

Diyan Li^1†^, Tiandong Che^1†^, Binlong Chen^1†^, Shilin Tian^1,2†^, Xuming Zhou^3†^, Guolong Zhang^4†^, Miao Li^1^, Uma Gaur^1^, Yan Li^1^, Majing Luo^5^, Long Zhang^1^, Zhongxian Xu^1^, Xiaoling Zhao^1^, Huadong Yin^1^, Yan Wang^1^, Long Jin^1^, Qianzi Tang^1^, Huailiang Xu^1^, Mingyao Yang^1^, Rongjia Zhou^5^, Ruiqiang Li^2^, Qing Zhu^1^ and Mingzhou Li^1^

^1^ Institute of Animal Genetics and Breeding, College of Animal Science and Technology, Sichuan Agricultural University, Chengdu, China

^2^ Beijing Novogene bioinformatics Technology Co., Ltd, Beijing, China

^3^ Division of Genetics, Department of Medicine, Brigham and Women’s Hospital, Harvard Medical School, Boston, USA

^4^ Department of Animal Science, Oklahoma State University, Stillwater, Oklahoma, USA

^5^ Hubei Key Laboratory of Cell Homeostasis, Laboratory of Molecular and Developmental Genetics, College of Life Sciences, Wuhan University, Wuhan, China

^†^ These authors contributed equally to this work.

**Correspondence:** [zhuqingsicau@163.com](mailto:zhuqingsicau@163.com); [mingzhou.li@sicau.edu.cn](mailto:mingzhou.li@sicau.edu.cn).

**Table S1.** A summary of the chickens used in this study: regions of collection/population and sequencing depths.

| Source | Breed | Location | Sample name | Group | Raw data(bp) | Effective ratio (%) | Mapping Ratio (%) | Depth (X) |
| --- | --- | --- | --- | --- | --- | --- | --- | --- |
| This study | Pengxian yellow fowl | Pengxian, Sichuan, 800m | LCPX1 | Cluster3 | 18,889,640,100 | 99.52 | 98.14 | 17.34 |
| This study |  |  | LCPX2 | Cluster3 | 19,278,011,700 | 99.66 | 98.42 | 17.81 |
| This study |  |  | LCPX3 | Cluster3 | 18,632,399,700 | 99.68 | 98.46 | 17.23 |
| This study |  |  | LCPX4 | Cluster3 | 15,280,677,900 | 99.56 | 98.29 | 14.09 |
| This study |  |  | LCPX5 | Cluster3 | 19,841,467,800 | 99.67 | 98.04 | 18.3 |
| This study |  |  | LCPX6 | Cluster3 | 17,335,756,800 | 99.79 | 98.07 | 16.02 |
| This study | Jinyang silky fowl | Jinyang, Sichuan, 460m | LCLS1 | Cluster2 | 18,049,413,000 | 99.7 | 98.87 | 16.8 |
| This study |  |  | LCLS2 | Cluster2 | 17,895,842,400 | 99.69 | 98.69 | 16.61 |
| This study |  |  | LCLS3 | Cluster2 | 15,627,352,800 | 99.58 | 97.32 | 14.2 |
| This study |  |  | LCLS4 | Cluster2 | 20,437,306,500 | 99.67 | 98.69 | 18.96 |
| This study |  |  | LCLS5 | Cluster2 | 17,792,258,100 | 99.69 | 98.37 | 16.43 |
| This study |  |  | LCLS6 | Cluster2 | 15,750,580,500 | 99.59 | 98.3 | 14.51 |
| This study | Emei black fowl | Leshan, Sichuan, 400m | LCEM1 | Cluster3 | 10,284,956,500 | 99.97 | 98.34 | 8.07 |
| This study |  |  | LCEM2 | Cluster3 | 32,475,811,500 | 99.99 | 95.39 | 25.52 |
| This study |  |  | LCEM3 | Cluster3 | 14,569,974,250 | 99.98 | 87.99 | 11.18 |
| This study |  |  | LCEM4 | Cluster3 | 54,438,319,250 | 96.84 | 98.9 | 44.05 |
| This study |  |  | LCEM5 | Cluster3 | 20,486,743,250 | 99.98 | 98.15 | 15.31 |
| This study |  |  | LCEM6 | Cluster3 | 10,707,922,250 | 99.98 | 98.44 | 8.51 |
| This study | Jiuyuan black-bone fowl | Yaan, Sichuan, 900m | LCJY1 | Cluster3 | 10,191,684,750 | 99.98 | 89.51 | 8.24 |
| This study |  |  | LCJY3 | Cluster3 | 41,435,362,750 | 99.99 | 94.08 | 31.96 |
| This study |  |  | LCJY5 | Cluster3 | 63,866,986,250 | 97.22 | 96.25 | 54.28 |
| This study |  |  | LCJY7 | Cluster3 | 16,269,159,300 | 99.63 | 98.36 | 15.46 |
| This study |  |  | LCJY8 | Cluster3 | 18,058,545,000 | 99.58 | 98.43 | 16.67 |
| This study | Muchuan black-bone fowl | Muchuan, Sichaun, 500m | LCMC1 | Cluster3 | 11,124,942,750 | 99.97 | 93.44 | 8.33 |
| This study |  |  | LCMC2 | Cluster3 | 18,076,646,250 | 99.97 | 98.44 | 13.88 |
| This study |  |  | LCMC4 | Cluster3 | 39,419,678,500 | 96.75 | 98.28 | 33.9 |
| This study |  |  | LCMC5 | Cluster3 | 14,012,295,250 | 99.97 | 98.92 | 10.96 |
| This study |  |  | LCMC8 | Cluster3 | 22,214,931,000 | 99.48 | 98.12 | 20.32 |
| This study | Miyi fowl | Panzhihua, Sihucan, 1400m | LCMY1 | Cluster2 | 10,438,894,750 | 99.98 | 96.33 | 8.49 |
| This study |  |  | LCMY2 | Cluster2 | 48,503,163,000 | 97.13 | 98.76 | 41.07 |
| This study |  |  | LCMY3 | Cluster2 | 32,281,322,250 | 99.99 | 97.96 | 24.69 |
| This study |  |  | LCMY4 | Cluster2 | 31,239,456,250 | 96.89 | 98.09 | 27.11 |
| This study |  |  | LCMY6 | Cluster2 | 9,281,997,250 | 99.97 | 93.88 | 7.43 |
| This study | Shimian caoke fowl | Shimian, Sichuan, 790m | LCSM4 | Cluster3 | 10,608,906,000 | 99.98 | 98.31 | 8.06 |
| This study |  |  | LCSM5 | Cluster3 | 14,371,852,500 | 99.98 | 94.17 | 11.05 |
| This study |  |  | LCSM6 | Cluster3 | 18,729,181,500 | 99.97 | 93.7 | 14.31 |
| This study |  |  | LCSM8 | Cluster3 | 17,811,655,200 | 99.57 | 97.82 | 16.27 |
| This study | Tianfu black-bone fowl | Chengdu, Sichuan, 540m, | LCTF2 | Cluster3 | 22,195,283,250 | 99.97 | 93.77 | 16.36 |
| This study |  |  | LCTF3 | Cluster3 | 66,147,003,500 | 96.63 | 99.03 | 56.97 |
| This study |  |  | LCTF4 | Cluster3 | 10,415,403,500 | 99.98 | 93.83 | 8.17 |
| This study |  |  | LCTF6 | Cluster3 | 45,393,903,000 | 99.99 | 98.16 | 34.17 |
| This study |  |  | LCTF8 | Cluster3 | 17,234,602,500 | 99.49 | 97.98 | 15.78 |
| This study | Tibetan fowl | Aba, Sichuan, 3300m | TCAB1 | Cluster1 | 10,011,460,500 | 99.97 | 92.32 | 8.17 |
| This study |  |  | TCAB3 | Cluster1 | 27,750,353,750 | 99.97 | 94.1 | 20.68 |
| This study |  |  | TCAB5 | Cluster2 | 10,093,982,000 | 99.97 | 91.39 | 8.29 |
| This study |  |  | TCAB6 | Cluster1 | 12,418,406,500 | 99.97 | 94.78 | 9.7 |
| This study |  |  | TCAB7 | Cluster3 | 18,225,233,700 | 99.59 | 98.11 | 16.73 |
| This study | Tibetan fowl | Diqing, Yunnan, 3280m | TCDQ1 | Cluster2 | 26,736,996,500 | 97.22 | 96.89 | 22.4 |
| This study |  |  | TCDQ2 | Cluster3 | 10,057,785,750 | 99.97 | 94.02 | 8.1 |
| This study |  |  | TCDQ3 | Cluster1 | 27,264,977,250 | 96.92 | 98.61 | 23.71 |
| This study |  |  | TCDQ4 | Cluster1 | 40,203,099,750 | 99.99 | 91.93 | 30.94 |
| This study |  |  | TCDQ5 | Cluster3 | 19,166,550,000 | 99.42 | 97.73 | 17.46 |
| This study |  |  | TCDQ6 | Cluster2 | 13,487,558,000 | 99.97 | 98.94 | 10.83 |
| This study | Tibetan fowl | Ganzi, Sichuan, 3390m | TCGZ1 | Cluster2 | 28,853,620,000 | 96.92 | 84.64 | 24.84 |
| This study |  |  | TCGZ3 | Cluster2 | 22,974,735,000 | 96.48 | 91.22 | 19.92 |
| This study |  |  | TCGZ4 | Cluster2 | 15,907,896,500 | 99.97 | 98.79 | 12.4 |
| This study |  |  | TCGZ5 | Cluster2 | 34,587,681,250 | 99.99 | 94.1 | 26.63 |
| This study |  |  | TCGZ6 | Cluster2 | 44,399,311,500 | 99.99 | 98.39 | 35.1 |
| This study |  |  | TCGZ10 | Cluster3 | 11,238,765,000 | 99.98 | 98.41 | 8.47 |
| This study | Tibetan fowl | Linzhi, Tibet, 3100m | TCLZ1 | Cluster3 | 10,396,378,500 | 99.97 | 88 | 8.22 |
| This study |  |  | TCLZ2 | Cluster1 | 12,685,078,750 | 99.97 | 97.69 | 9.79 |
| This study |  |  | TCLZ3 | Cluster1 | 10,219,404,500 | 99.98 | 98.14 | 7.85 |
| This study |  |  | TCLZ4 | Cluster2 | 23,333,864,000 | 99.97 | 83.49 | 17.15 |
| This study |  |  | TCLZ5 | Cluster3 | 21,372,917,000 | 99.99 | 94.1 | 17.08 |
| This study | Tibetan fowl | Haiyan, Qinghai, 3260m | TCQH1 | Cluster2 | 13,908,831,500 | 98.06 | 93 | 7.97 |
| This study |  |  | TCQH11 | Cluster1 | 14,309,430,750 | 99.97 | 94.28 | 11.51 |
| This study |  |  | TCQH10 | Cluster1 | 19,186,282,200 | 99.36 | 97.88 | 17.47 |
| This study |  |  | TCQH5 | Cluster1 | 13,076,345,500 | 99.98 | 97.72 | 9.11 |
| This study |  |  | TCQH8 | Cluster1 | 19,061,293,500 | 99.43 | 97.7 | 17.39 |
| This study |  |  | TCQH9 | Cluster1 | 18,761,834,100 | 99.47 | 97.85 | 17.14 |
| This study | Tibetan fowl | Shannan, Tibet, 3700m | TCSN1 | Cluster1 | 28,838,655,500 | 97.01 | 93.4 | 25.03 |
| This study |  |  | TCSN3 | Cluster3 | 32,232,808,000 | 99.99 | 98.17 | 25.33 |
| This study |  |  | TCSN4 | Cluster3 | 24,875,402,750 | 97.08 | 98.6 | 21.73 |
| This study |  |  | TCSN5 | Cluster3 | 26,483,858,000 | 99.99 | 92.93 | 20.41 |
| This study |  |  | TCSN6 | Cluster1 | 13,123,978,750 | 99.97 | 98.71 | 10.49 |
| This study |  |  | TCSN7 | Cluster1 | 14,802,134,400 | 99.75 | 97.95 | 13.64 |
| This study |  |  | TCSN8 | Cluster1 | 16,959,536,100 | 99.72 | 98.52 | 15.72 |
| This study |  |  | TCSN9 | Cluster1 | 15,271,966,500 | 99.39 | 97.5 | 13.87 |
| NCBI | Xishuangbanna game fowl | Xishuangbanna, Yunnan, 1500m | LCYN1 | Cluster1 | 19,844,288,800 | 96.07 | 98.28 | 17.45 |
| NCBI |  |  | LCYN2 | Cluster1 | 12,417,572,000 | 95.34 | 98.16 | 10.9 |
| NCBI |  |  | LCYN3 | Cluster1 | 19,152,411,186 | 94.57 | 98.84 | 16.49 |
| NCBI |  |  | LCYN4 | Cluster1 | 36,653,630,400 | 51.44 | 98.39 | 17.28 |
| NCBI |  |  | LCYN5 | Cluster1 | 20,524,055,400 | 94.77 | 98.76 | 17.72 |
| NCBI |  |  | LCYN6 | Cluster1 | 22,299,766,600 | 97.18 | 98.9 | 19.51 |
| NCBI |  |  | LCYN7 | Cluster1 | 16,772,316,000 | 92.19 | 96.25 | 13.36 |
| NCBI |  |  | LCYN8 | Cluster1 | 30,481,395,600 | 70.66 | 99.03 | 19.71 |
| NCBI | Red jungle fowl | Yunnan | RJF1 | Cluster1 | 17,338,095,736 | 89.8 | 98.29 | 14.08 |
| NCBI |  |  | RJF2 | Cluster1 | 28,604,283,000 | 97.93 | 88.96 | 24.66 |
| NCBI |  |  | RJF3 | Cluster1 | 18,084,321,682 | 92.64 | 93.41 | 15.39 |
| NCBI |  |  | RJF5 | Cluster1 | 40,234,827,200 | 96.19 | 98.84 | 34.95 |
| NCBI |  | Hainan | RJF4 | Cluster1 | 21,713,560,968 | 94.42 | 94.65 | 18.66 |

Note: Cluster1, Cluster2 and Cluster3 are correspond to Cluster1, Cluster2 and Cluster3 respectively in Figure 1 and Figure 2.

| **Table S2.** SNPs annotation and genetic diversity of 17 chicken populations analyzed in this study. | | | | | | | | | | | | | | |  |  |  |
| --- | --- | --- | --- | --- | --- | --- | --- | --- | --- | --- | --- | --- | --- | --- | --- | --- | --- |
| Type | Population (n) | Total SNP (Mb) | Upstream (kb) | Exonic | | | | | Intronic (Mb) | Splicing (bp) | Intergenic (Mb) | Upstream/Downstream | Downstream (kb) | *θ*π(×10^-3^) | *θ*ω(×10^-3^) | *θ*π(×10^-3^) | *θ*ω(×10^-3^) |
|  |  |  |  |  |  |  |  |  |  |  |  |  |  |  |  |  |  |
|  |  |  |  | Nonsynonymous (kb) | Synonymous (kb) | Nonsyn/Syn ratio(ω) | Stop gain | Stop loss |  |  |  |  |  |  |  |  |  |
| Sichuan local  chicken breeds | Emei(6) | 6.60 | 128.13 | 28.66 | 78.88 | 0.36 | 208 | 27 | 2.49 | 246 | 3.77 | 5.21 | 92.98 | 2.44 | 2.17 | 2.43 | 2.03 |
|  | Jiuyuan(5) | 6.51 | 123.40 | 27.75 | 76.90 | 0.36 | 199 | 27 | 2.46 | 253 | 3.72 | 4.98 | 90.66 | 2.47 | 2.27 |  |  |
|  | Jinyang(6) | 6.63 | 124.97 | 28.11 | 78.28 | 0.36 | 211 | 29 | 2.51 | 253 | 3.80 | 4.94 | 92.30 | 2.40 | 2.16 |  |  |
|  | Muchuan(5) | 6.69 | 128.14 | 28.82 | 79.61 | 0.36 | 216 | 27 | 2.53 | 250 | 3.82 | 5.12 | 93.13 | 2.54 | 2.35 |  |  |
|  | Miyi(5) | 5.27 | 100.85 | 22.09 | 62.14 | 0.36 | 150 | 23 | 1.99 | 215 | 3.02 | 4.11 | 73.51 | 1.90 | 1.66 |  |  |
|  | Pengxian(6) | 6.25 | 118.71 | 26.55 | 73.62 | 0.36 | 191 | 30 | 2.37 | 226 | 3.58 | 4.79 | 87.28 | 2.29 | 2.01 |  |  |
|  | Shimian(4) | 5.63 | 109.51 | 24.36 | 67.82 | 0.36 | 170 | 23 | 2.12 | 223 | 3.22 | 4.44 | 79.18 | 2.22 | 2.05 |  |  |
|  | Tianfu(5) | 6.74 | 128.27 | 28.85 | 79.83 | 0.36 | 203 | 30 | 2.54 | 246 | 3.86 | 5.15 | 93.87 | 2.57 | 2.38 |  |  |
| Tibetan chicken  populations | Aba(5) | 6.69 | 127.84 | 28.63 | 79.98 | 0.36 | 195 | 27 | 2.53 | 252 | 3.83 | 5.23 | 93.31 | 2.55 | 2.36 | 2.63 | 2.02 |
|  | Diqing(6) | 7.28 | 139.20 | 31.65 | 86.73 | 0.36 | 224 | 34 | 2.75 | 277 | 4.16 | 5.63 | 101.82 | 2.66 | 2.44 |  |  |
|  | Ganzi(6) | 6.93 | 133.24 | 29.98 | 82.44 | 0.36 | 213 | 30 | 2.62 | 260 | 3.96 | 5.38 | 96.88 | 2.51 | 2.28 |  |  |
|  | Linzhi (5) | 6.74 | 129.47 | 29.30 | 80.88 | 0.36 | 211 | 28 | 2.54 | 255 | 3.86 | 5.32 | 94.23 | 2.58 | 2.38 |  |  |
|  | Haiyan(6) | 7.05 | 135.27 | 30.34 | 84.37 | 0.36 | 219 | 30 | 2.67 | 268 | 4.03 | 5.47 | 99.09 | 2.58 | 2.35 |  |  |
|  | Shannan(8) | 7.52 | 143.17 | 32.42 | 89.06 | 0.36 | 228 | 38 | 2.84 | 291 | 4.30 | 5.82 | 104.61 | 2.62 | 2.30 |  |  |
| Xishuangbanna game fowl | Xishuangbanna (8) | 7.11 | 134.38 | 29.83 | 83.75 | 0.36 | 212 | 31 | 2.70 | 265 | 4.06 | 5.40 | 98.95 | 2.50 | 2.18 | 2.50 | 2.18 |
| Red jungle fowl | RJF_Hainan(1) | 3.46 | 60.20 | 12.66 | 39.57 | 0.32 | 85 | 12 | 1.32 | 123 | 1.96 | 2.35 | 46.17 | - | - | - | - |
|  | RJF_Yunnan(4) | 6.37 | 116.33 | 24.53 | 72.87 | 0.34 | 169 | 24 | 2.41 | 236 | 3.60 | 4.65 | 86.19 | 2.58 | 2.44 | 2.58 | 2.44 |

Note: ‘Upstream’ refers to a variant that overlaps with the 1-kb region upstream of the gene start site. ‘Stop gain’ indicates that a nonsynonymous SNP leads to the creation of a stop codon at the variant site. ‘Stop loss’ indicates that a nonsynonymous SNP leads to the elimination of a stop codon at the variant site. ‘Splicing’ indicates that a variant is within 2-bp of a splice junction. ‘Downstream’ indicates that a variant overlaps with the 1-kb region downstream of the gene end site. ‘Upstream/Downstream’ indicates that a variant is located in the downstream and upstream regions (possibly for two different genes). (*θw*): average nucleotide polymorphism. (*θπ*): nucleotide diversity. Emei: Emei black fowl; Jiuyuan: Jiuyuan black fowl; Jinyang: Jinyang silky fowl; Muchuan: Muchuan black-bone fowl; Miyi: Miyi fowl; Pengxian: Pengxian yellow fowl; Shimian: Shimian caoke fowl; Tianfu: Tianfu black-bone fowl; Tibetan populations include Aba, Ganzi, Shannan, Linzhi, Diqing and Haiyan; RJF: red jungle fowl; Game fowl: Xishuangbanna game fowl (Table S1).

**Table S3.** The general phenotypic differences between RJF, Tibetan and Sichuan local chickens.

| Breed | Red jungle fowl | Tibetan fowl | Miyi fowl | Emei black fowl | Muchuan black-bone fowl | Shimian caoke fowl | Tianfu black-bone fowl | Jinyang silky fowl | Pengxian yellow fowl | Jiuyuan black-bone fowl |
| --- | --- | --- | --- | --- | --- | --- | --- | --- | --- | --- |
| Place of origin | Yunnan | Qinghai-Tibet Plateau | Szechwan Basin | Szechwan Basin | Szechwan Basin | Szechwan Basin | Szechwan Basin | Szechwan Basin | Szechwan Basin | Szechwan Basin |
| Colour (♂) | Golden, red, black blue | Golden,red, black blue | Red, black | Black, reddish black | Black | Black, red | Black | White | Yellow, red, black | Black, reddish black |
| Skin colour (♂) | White | White | White | White | Corvinus | White | Corvinus | White | White | Corvinus |
| Birth weight (♂) (g) | 26 | 30 | — | 40 | — | 41 | 31 | — | 40 | — |
| Sexual maturity (♂) | 120 | 120 | 120 | — | 200 | 208 | — | 120 | 120 | — |
| Age at first egg (♀) (day) | 252.27 | 232.06 | 191.85 | 179.28 | 187.47 | 157.45 | 148.76 | 156.73 | 169.54 | 181.32 |
| Body weight at first egg (♀) (g) | 860.68 | 1120.45 | 1793.67 | 1896.52 | 2194.14 | 2215.77 | 1423.62 | 1195.84 | 1438.86 | 1582.29 |
| Egg number at 300 days of age (♀) | 22.52 | 42.75 | 57.29 | 83.34 | 78.25 | 98.93 | 102.46 | 67.57 | 91.69 | 73.63 |
| Egg weight at 300 days of age (♀) (g) | 29.15 | 34.97 | 54.35 | 53.81 | 54.03 | 55.43 | 55.43 | 52.47 | 54.52 | 55.62 |
| Body weight at 180 days of age (♂) (g) | 1113.4 | 1225.22 | 2178.06 | 2642.3 | 2484.3 | 3278.25 | 2485.67 | 2090 | 2032.35 | 2238.81 |
| Body weight at 180 days of age (♀) (g) | 780.65 | 1085.52 | 1517.65 | 1889.5 | 2031.86 | 2727.95 | 1594.51 | 1340.45 | 1524.28 | 1567.06 |
| Whole net carcass rate (♂) (%) | — | 73.28 | 77.7 | 79.65 | 78.9 | 69.5 | — | — | 79.1 | 79.7 |
| Whole net carcass rate (♀) (%) | — | 72.17 | 70.4 | 70.9 | 69.4 | 65.2 | — | — | 72.12 | 67 |
| Adult weight (♂) (g) | — | 1490 | 2405 | 2622 | 2680 | 3650 | — | 2230 | 3950 | 2615.5 |
| Adult weight (♀) (g) | — | 1150 | 1985 | 1904 | 2290 | 3060 | — | 1450 | 1880 | 1762.5 |
| Body slanting length (♂) (cm) | — | 21.2 | 25.62 | 26.3 | 28.2 | 25.4 | — | 24.9 | 24.4 | 28.8 |
| Body slanting length (♀) (cm) | — | 19.2 | 23 | 22.1 | 18.22 | 22.4 | — | 21.1 | 20.5 | 24.2 |
| Chest breadth (♂) (cm) | — | 7.2 | 9.19 | 8.5 | 9.08 | 9.6 | — | 8.6 | 8.1 | 9.4 |
| Chest breadth (♀) (cm) | — | 7.01 | 7.31 | 6.9 | 7.44 | 8.7 | — | 6.5 | 6.7 | 8 |
| Chest depth (♂) (cm) | — | 6.4 | 11.6 | 12.6 | 9.02 | 11.2 | — | 11.7 | 11.9 | 11.8 |
| Chest depth (♀) (cm) | — | 6 | 10.72 | 10.3 | 8.54 | 8.7 | — | 10.1 | 9.9 | 11 |
| Pelvis width (♂) (cm) | — | 7.6 | 8.46 | 9.6 | 9.08 | 11.3 | — | 8.7 | 9.9 | 9.6 |
| Pelvis width (♀) (cm) | — | 7.3 | 7.88 | 8.3 | 8.18 | 11.1 | — | 7.7 | 8.2 | 8.72 |
| Shank length (♂) (cm) | — | 9.5 | 12.21 | 13.9 | 11.5 | 14.8 | — | 12 | 12.3 | 13.1 |
| Shank length (♀) (cm) | — | 8.5 | 9.9 | 10.1 | 8.99 | 11.3 | — | 9.1 | 9.5 | 10.6 |

Note: Body slanting length, chest breadth, chest depth, Pelvis width and shank length traits are all corresponding to adults. All phenotypic data were collected from references or Sichuan Local Varieties. “—” indicate unavailable.

**Figure S1.** Average nucleotide polymorphism (*θ*_ω_) and nucleotide diversity (*θ*_π_) among Sichuan local chickens, Tibetan chickens and red jungle fowls.


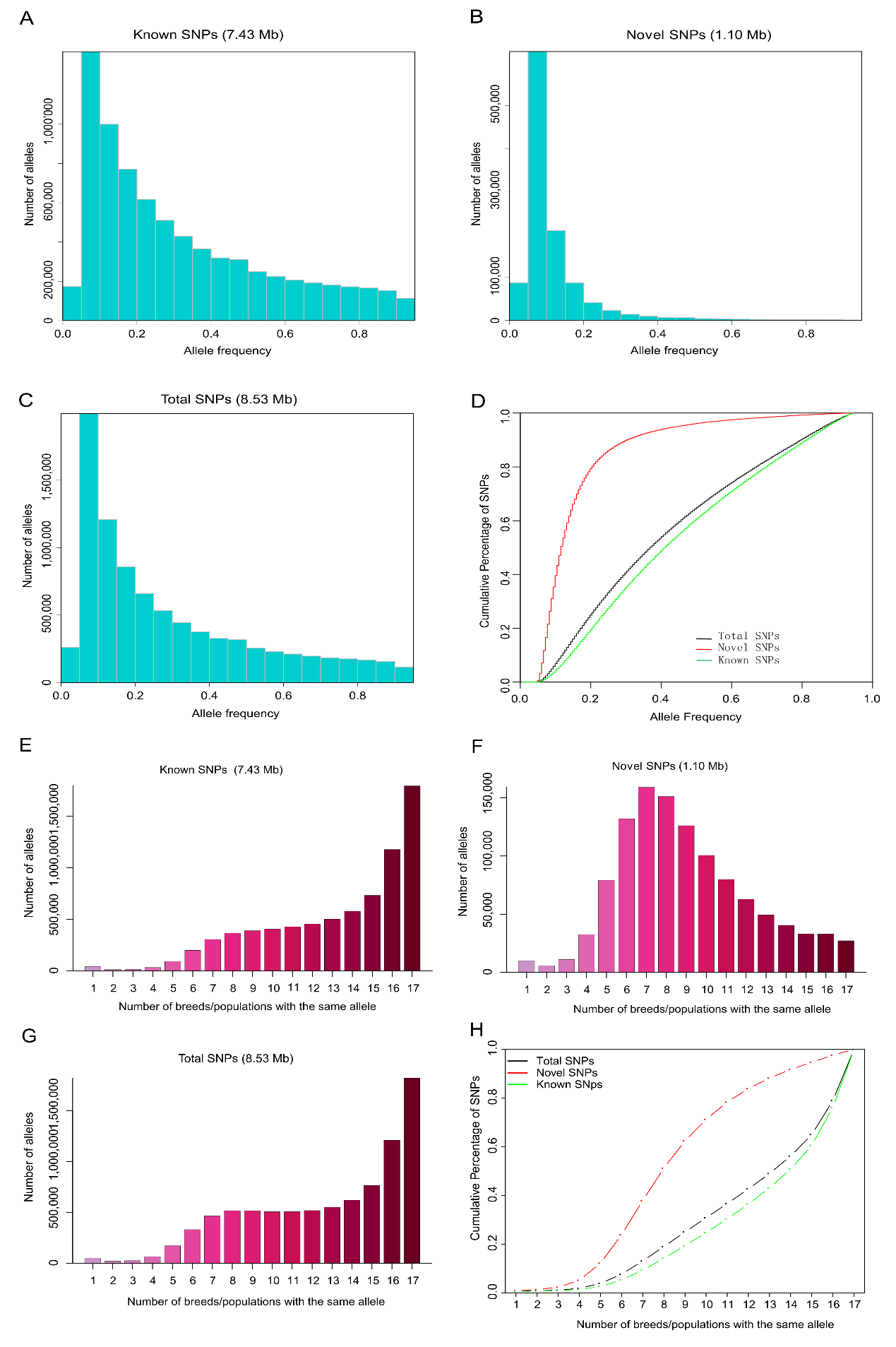


**Figure S2.** Allele frequency spectra in 91 birds and Number of alleles distribute in 1 to 17 chicken breeds/populations. Panel A, B and C represent distribution of allele frequency of known, novel and total SNPs in 91 birds respectively. Panel D and H is accumulative percentage of SNPs. Panel E, F and G represent distribution of alleles of known SNPs, novel SNPs and total SNPs in 1 to 17 chicken breeds/populations respectively.


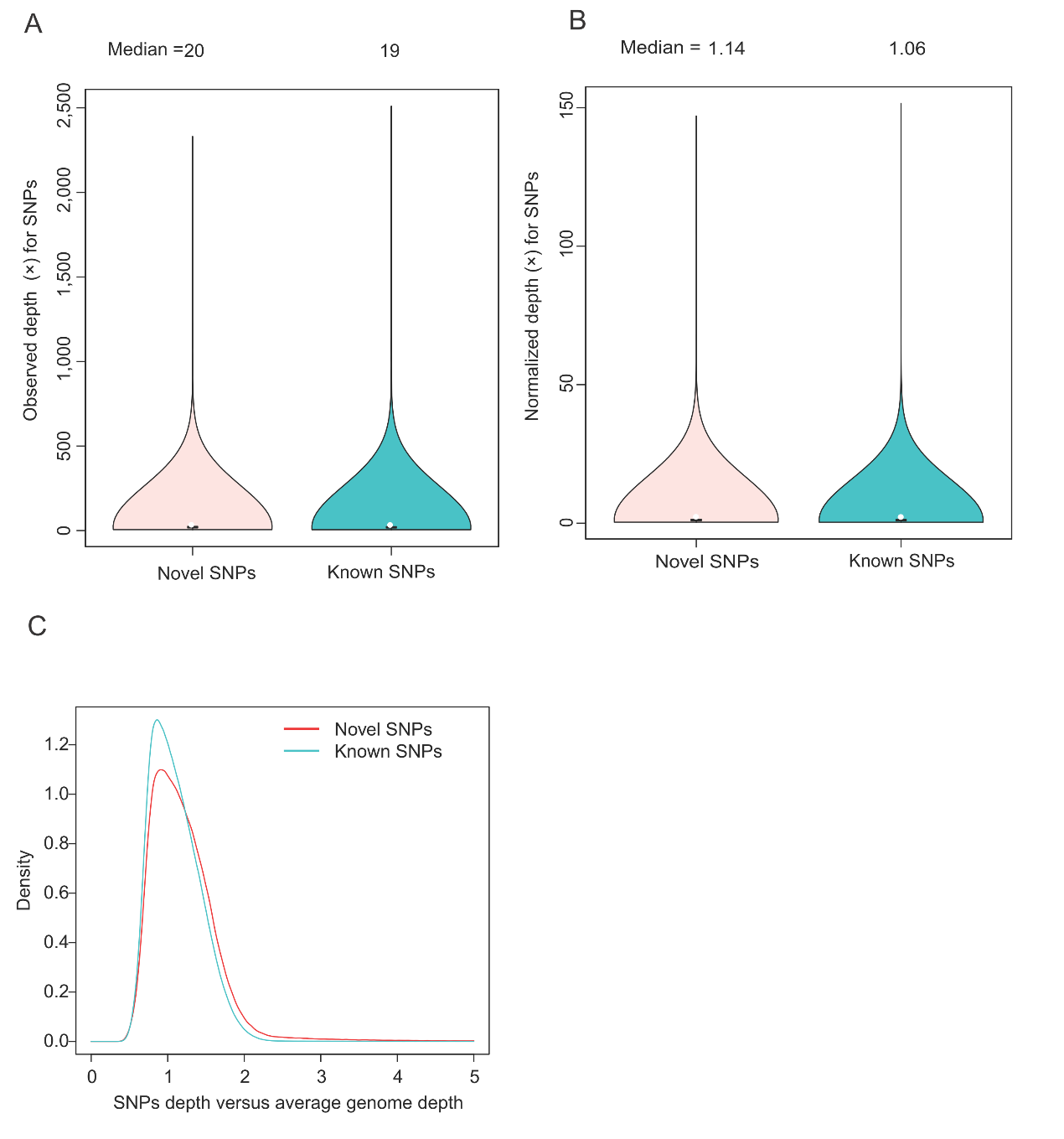


**Figure S3.** Comparison of read depth between the known and novel SNPs. Violin plots of observed depth for SNPs (A) and SNPs depth versus average genome depth (B); density distribution of SNPs depth versus average genome depth (C).


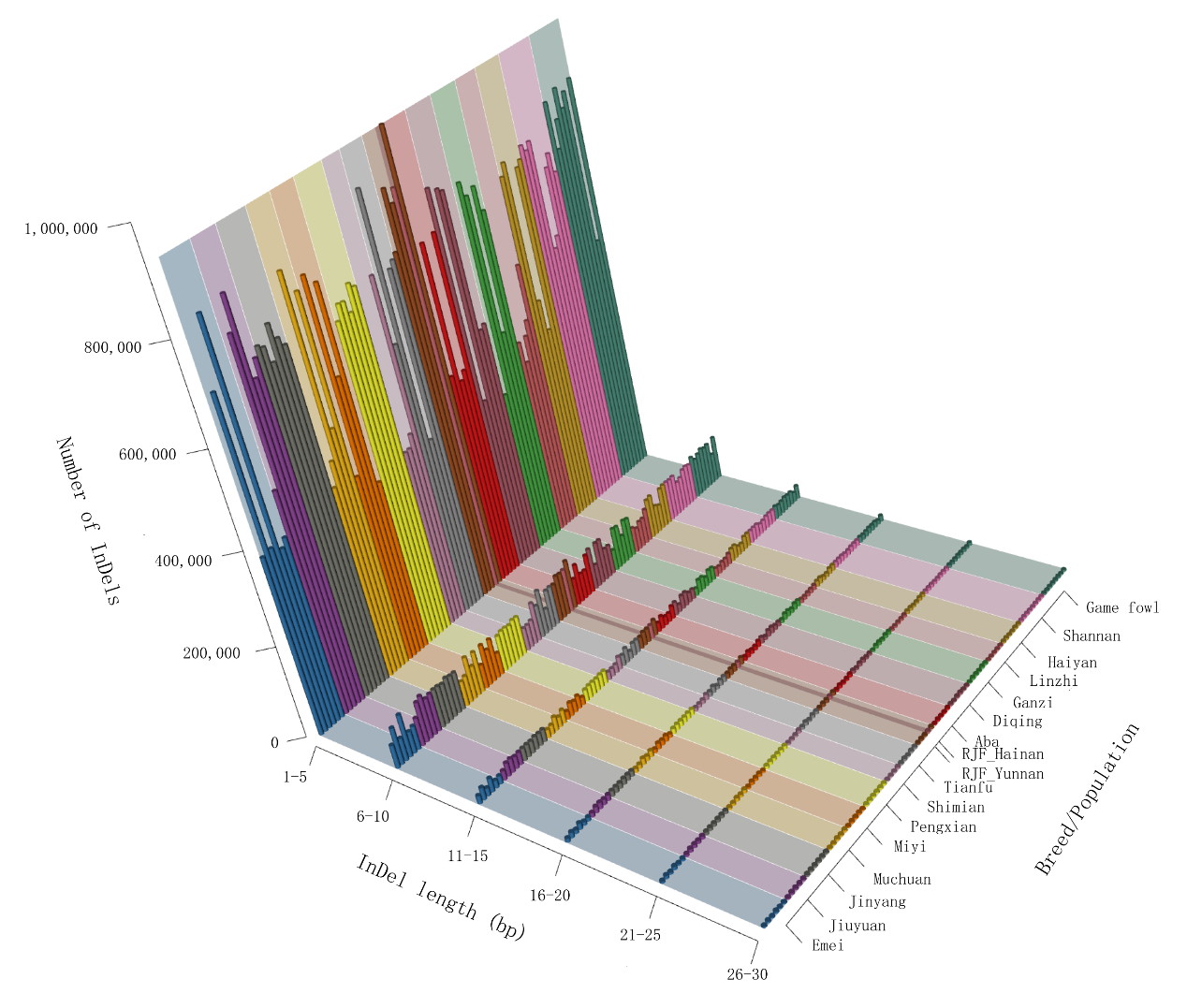


**Figure S4.** Overall distribution of the lengths of InDels (1-30 bp).


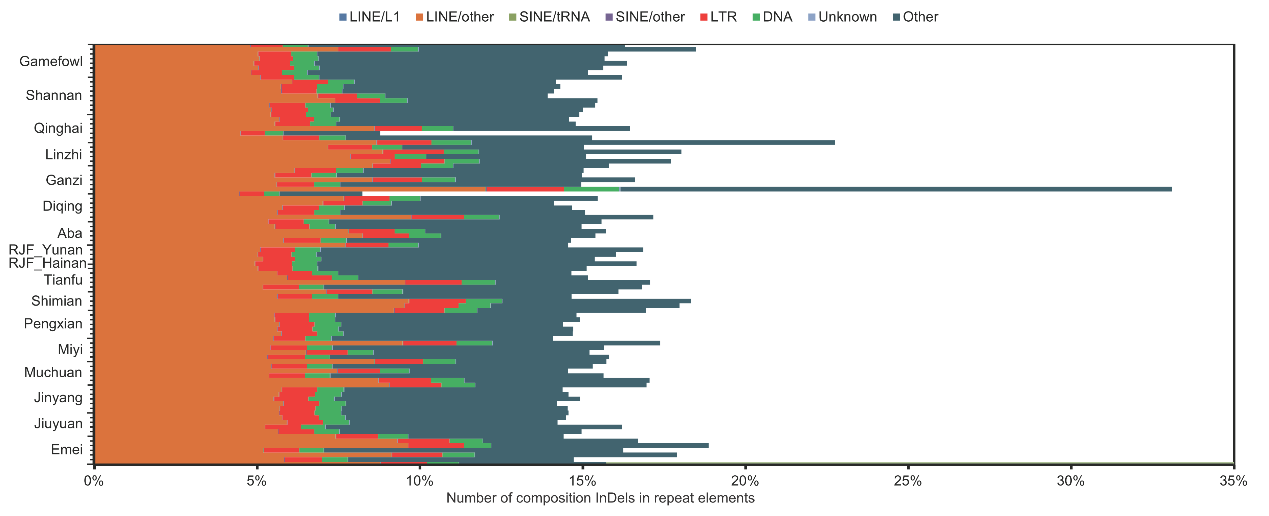


**Figure S5.** Percentage composition of InDels in repeat elements.





**Figure S6.** Percentage distribution (A) and probability (B) for InDels across different genomic elements.


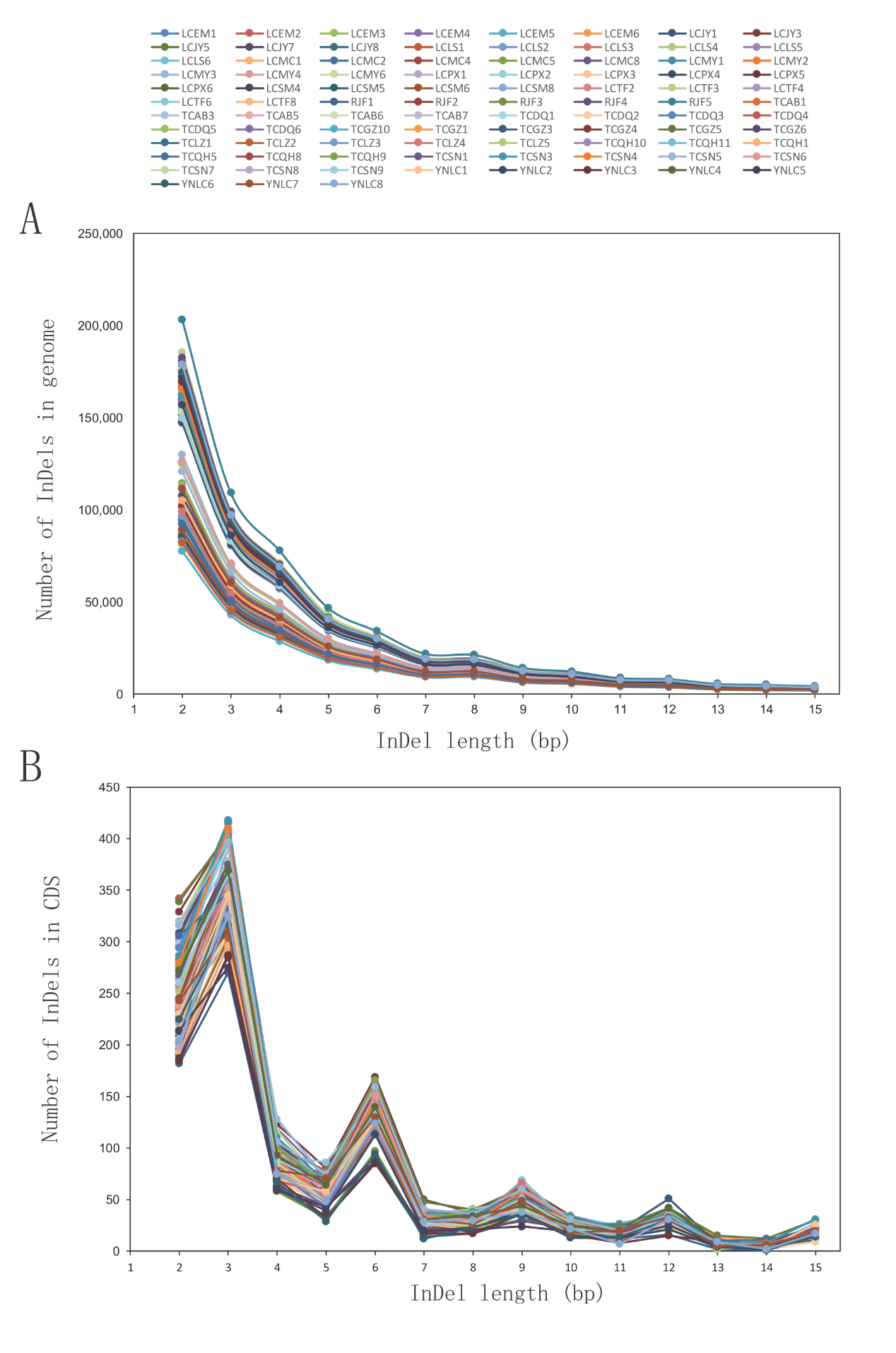


**Figure S7.** Length distribution of small InDels in the whole genome (A) and coding sequence (CDS) regions (B).
