# Supplementary material for: Genomic data for 78 chickens from 14 populations
Source: Gigascience. 2017 Apr 18;6(6):1–5. doi: 10.1093/gigascience/gix026 (PMC5449643; doi:10.1093/gigascience/gix026)
Supplement: Response_to_reviewer_comments_Revision_2.pdf [file gix026_Response_to_reviewer_comments_Revision_2.pdf]

Dear Dr. Hans Zauner,
We are delighted to be informed of the positive responses from you. We have carefully revised the manuscript following reviewer 1's suggestions and provide a copy of reviewers' comments with our point-to-point responses, and upload it online for formal acceptance and publication.
We sincerely appreciate your assistance in improving the manuscript. We are glad to be able to contribute to GigaScience.
Best regards,

Detailed responses to reviewers
Reviewer #1:
Comment 1-1:
In the revised manuscript, the authors have sufficiently addressed most of my earlier comments.
Response 1-1:
We sincerely appreciate your comments in improving the manuscript.

Comment 1-2:
The only problem I still have is in the choice of the clustering (1-3) as suggested by the authors. In particular, the grouping of the animals in cluster two seems odd when one looks at the result of the PCA analyses. I think it would make more sense to split cluster 2 into two clusters. The first dimension of a PCA represents the largest difference seen between the individuals and cluster 2 actually consist of two clusters (2L and 2R) that are equally distant to each other as 2R is to cluster 3. I fail to see the arguments why the authors still group all of these into a single cluster.
Response 1-2:
Thank you for this thoughtful comment. Although the branches in the NJ tree are not very easy to see, we agree that it is more appropriate to fur¬ther split the cluster 2 into two sub-clusters in view of the result of the PCA analyses.
We have carefully added the corresponding text as below:
'The principal component analysis (PCA) as implemented in EIGENSOFT package recapitulated these findings (Figure 2B) and revealed that the cluster 2 can be further split into two sub-clusters. The Tibetan fowls in cluster 2 are more genetically close to the Jinyang silky fowls (sub-cluster 2-R) than Miyi fowls (sub-cluster 2-L) (Figure 2B).'
We hope this amendment will give the reader a more comprehensive understanding of relatedness among populations.
Comment 1-3:
The term overlapped SNPs seems a bit odd. I suggest to refer to these as "known SNPs". The contrast known vs novel seems more appropriate.
Response 1-3:
Changed as suggested.

Comment 1-4:
Line 144: (at least in an individual) replace "an" by "one"
Response 1-4:

Changed as suggested.

Comment 1-5:
I am confused by the "normalized depth" in line 145 (median of normalized depth of 1.14 versus 1.06). The authors state that the novel SNPs also exhibited a comparable sequencing depth with the overlapped SNPs (median of normalized depth of 1.14 versus 1.06). I probably misunderstand this term but could the authors explain what this means (given that the average sequencing depth is 18).
Response 1-5:
We acknowledge reviewer 1's comments. The 'normalized read depth of SNPs' was defined as SNP depth versus average genome depth. To avoid this ambiguous description, we have carefully rewrote the statement as follows in the main text and revised the corresponding labels and legends for Figure S3.
'These novel SNPs exhibited a similar read depth with the known SNPs (median of 20 × versus 19×), which are both comparable with the average depth for the genome (median of 1.14-fold versus 1.06-fold) (Additional file 1: Figure S3).'
