# Supplementary material for: Genomic data for 78 chickens from 14 populations
Source: Gigascience. 2017 Apr 18;6(6):1–5. doi: 10.1093/gigascience/gix026 (PMC5449643; doi:10.1093/gigascience/gix026)
Supplement: Response_to_reviewer_comment_Revision_1.pdf [file gix026_Response_to_reviewer_comment_Revision_1.pdf]

Dear Hans Zauner,

Thank you very much for processing our manuscript entitled “Genomic data for 78 chickens from 14 populations " (GIGA-D-16-00092R1) for possible publication in GigaScience. We are very grateful to you and three reviewers for their helpful suggestions.

Detailed responses to reviewers

Reviewer #1

In their revised manuscript the authors have addressed many, but not all of my previous comments. Unfortunately, most of that information is buried in the supplementary material, and not discussed or referred to in the manuscript. The authors only discuss this in their reply to the comments of the reviewers. I think it is essential that the authors describe these issue in the paper itself. They should not only satisfy the reviewers, but should make the paper itself more clear to future readers.

Thanks for the reviewer’s helpful suggestion. As suggested, we have discussed these issues in the manuscript.

Comment 1-1:

I think the authors have misunderstood my comment about the allele frequency spectra. I meant a plot that shows the number of SNPs for different MAF (minor allele frequency) classes. This is related to my previous comment number 3. Adding a plot for the SNPs that are already in dbSNP and the novel SNPs identified in this study should also show a clear difference (most novel ones have very low MAF).

Response 1-1:

Thanks for your suggestion. We have added a plot for the SNPs that are already in dbSNP and the novel SNPs identified in this study (Figure S2). As expected, most novel ones (89.02%) had low allele frequency (<0.2) compared with total (49.91%) and known SNPs (44.02%).

Comment 1-2:

Related to the low MAF of the novel SNPs: I think the authors should comment on the false discovery rate of the SNPs or at least indicate that this is to be expected to be higher for SNPs with low MAF (so higher in the novel ones).

Response 1-2:

Thanks for your comment and helpful suggestions. We are sorry for not providing FDR. But we added other additional analysis to estimate the properties of these novel SNPs, and the differences in many parameters between known and the novel SNPs. In our original version of this manuscript, we have indicated that in order to get highly credible SNPs, we used strict criteria with filtering MAF (minor allele frequency) < 0.05 and missing genotype > 10% in chicken population and obtained 8.53 Mb SNPs. These 8.53 Mb highly credible SNPs were then classified to known and novel SNPs.

Line 141-148: As shown in Additional file 1: Figure S2, although most novel SNPs (89.02%) had a low allele frequency (<0.2 of 91 individuals) compared with the overlapped SNPs (44.02%), only 9,918 (0.88% of 1.10 M) novel SNPs were specifically detected in one breed/population (at least in an individual). These novel SNPs also exhibited a comparable sequencing depth with the overlapped SNPs (median of normalized depth of 1.14 versus 1.06) (Additional file 1: Figure S3). In addition, we observed more than 75% of the novel SNPs and

86% of the overlapped SNPs were in non-repeat regions. These results suggest the novel SNPs will serve as a potentially valuable resource for further chicken studies.
Because we found a difference of allele frequency distribution between the known and novel SNPs (Figure S2 and S3), we further checked the mean depth, missing rate of SNPs in 91 individuals, distance of SNP to closest gapped region, distance with neighbor SNPs, percentage of GC content (100 bp upstream and downstream to SNP), percentage of gapped region content (100 bp upstream and downstream to SNP), percentage of repeat content (100 bp upstream and downstream to SNP), percentage of gapped region content (1000 bp upstream and downstream to SNP) and percentage of repeat content (1000 bp upstream and downstream to SNP). There is no difference in these parameters between the known and novel SNPs (https://www.dropbox.com/s/avztcx3xksher6v/known%20vs%20novel%20SNPs.pdf?dl=0).
We also checked the allele distribution in 17 chicken breeds/populations. The results showed different distribution pattern of known and novel SNPs. In novel SNPs, only 9,918 alleles (0.88%) were detected in just one breed/population. The remaining alleles were supported by two or more than two breeds/populations. Most novel SNPs were detected in small numbers of breeds/populations (≤9 breeds/populations, 62.39% of SNPs) compared with known SNPs (19.36% of SNPs).

Comment 1-3:
I still do not agree with the grouping of the animals into 7 distinct clusters. The branches in the NJ tree are not very easy to see, but the tree nevertheless suggests that:
- SW2 and TC2 form 1 cluster consisting of 4 sub-clusters.
- Two of the RJF seem to cluster with SW3
- SW1 and TC3 cluster together into a single large cluster. If one would make separate sub-clusters, the TC3 individuals would be split into two subgroups together with different SW1 individuals.

This is also confirmed by the PCA plot, where SW2 and TC2 cluster together or form two separate clusters: (Cluster 1: 5 SW2 and 1 TC2 and cluster 2 with 6 SW2 and the remaining TC2 individuals. Likewise, SW1 and TC3 can be considered as 1 cluster (or two highly related populations with a bit of overlap).
Response 1-3:
Thanks for your helpful suggestion, we agree with you the grouping of the animals into 3 distinct clusters. Relative changes have been made in our revised MS (Figure 2).

Comment 1-4:
The abbreviations SW, TC are still not defined in the manuscript. The authors have added information about small indels to the supplement. However this is not mentioned in the manuscript. (also they still do not clearly mention in the manuscript that the numbers all refer to substitutions).
Response 1-4:
The abbreviations SW, TC were removed from our new MS. We have added more description of InDels in the revised MS.
Line 149-158 (InDels description): Overall distribution of the lengths of insertions and deletions (InDels) showed that more than 80% of the InDels were 1-5 bp in length (Additional file 1: Figure S4). Repetitive elements (10.61% of the genome and containing ~15.70% of InDels) are an important source of structural variation in chicken genome (Additional file 1: Figure S5).

About a half of InDels (48.39% to 51.52%) were occurred in the intergenic regions (588.65 Mb and 56.23% of the genome). The introns (403.35 Mb and containing ~43.86% of InDels) showed higher incidence of InDels than the coding sequences (25.81 Mb and containing ~1.77% of InDels) (Additional file 1: Figure S6). We observed an enrichment of short InDels (1-15 bp in length) in coding sequences that were multiples of 3 bp compared to whole genome sequences, which is expected to preserve the reading frame (Additional file 1: Figure S7).
Comment 1-5:
The novel SNPs should be submitted to dbSNP and accession numbers should be provided in the manuscript.
Response 1-5:
The 1.10 Mb novel SNPs had been submitted to dbSNP and accession numbers are provided in Additional file 2. The SNPs were linked to our project ID (PRJNA306389).

Reviewer #2: The authors have addressed all of my original comments and I believe this has improved the readability of the manuscript.
As a result, I am happy to support the progression of this manuscript to publication.
Thanks for your positive comments and revising our MS.

Reviewer #3: The authors' response clarifies most of my original points.
Comment 3-1:
Table 1 was a neat addition to figure 1, but now when it's clearer I'm not sure I understand the meaning of the dashed circles (around the populations) in Fig 1A anymore. In the original figure the circles corresponded to the group(s), but in the revised figure the label TC1 has been removed from the left-most circle, and from Table 1 I understand that those three populations (13, 14, 15) actually contain individuals from all three TC groups - so maybe this circle can be removed altogether?
The colors helped me a lot when connecting the different parts of the figure, but personally I think it would be even clearer if also the numbers in Fig 1A could be repeated, preferably also in Table 1 (as it is now one need to go via the Figure text to connect Fig 1A and Table 1).
Response 3-1:
Thanks for your comment, in order to make sense, we have combined table 1 and figure 1 in our new MS and removed the repetition.

Comment 3-2:
The additional file now also contains figures, but I can't view two of them (in Table S2 and the lower one in Table S5), it looks like the links are broken. The top figure in Table S5 lacks text in the color legend. Also I think the figures should be given separate names (Figure S1, S2 etc) so they can be referred to properly in the text.
Response 3-2:
We have made relative changes in our revised MS, all figures and tables were provided separately.

Comment 3-3:
The new supplementary table S4 (referred to as "Allele frequency spectrum" both on page 4, line 124 and on page 6, line 189) doesn't seem to contain the Allele frequency spectrum but rather a summary of the types of SNPs for each individual.

Response 3-3:
Thanks for your careful review. Allele frequency spectrum were provided in Figure S2 in our new MS, and the supplementary table S4 is removed.

Comment 3-4:
Minor comments:
Page 1, line 28: change "gigabase (Gb) high quality data of" to "gigabases (Gb) of high quality data from"
Response 3-4:
As suggested, "gigabase (Gb) high quality data of" was changed to "gigabases (Gb) of high quality data from".

Comment 3-5:
Page 1, line 43: "base resequencing data" should be "bases of resequencing data". On a personal note I don't think I've seen the word "trillion" in a scientific text before and I think I would prefer to write it as *10^12, but of course it's not wrong as it is now.
Response 3-5:
As suggested, " base resequencing data " was changed to " bases of resequencing data ".

Comment 3-6:
Page 2, line 58: change "by following steps" to "by the following steps"
Response 3-6:
We have changed "by following steps" to "by the following steps".

Comment 3-7:
Page 2, line 71: change "we further used command" to "we further used the command"
Response 3-7:
"we further used command" was changed to "we further used the command".

Comment 3-8:
Page 4, line 123-124: the sentence "Among them, there was 1,812,591 (21.24%) SNPs could be detected" needs to be corrected, maybe remove "there was"?
Response 3-8:
"there was" was deleted in our revised MS.

Comment 3-9:
Page 4, line 125: "was presented" -maybe use present tense when referring to table/figure?
Response 3-9:
"was presented" was changed to “are present”.

Comment 3-10:
Table S4: Header of column H should be changed from "No validated" to "Not validated".
Response 3-10:
Table S4 has been removed from our revised MS.
