# Supplementary material for: Genomic data for 78 chickens from 14 populations
Source: Gigascience. 2017 Apr 18;6(6):1–5. doi: 10.1093/gigascience/gix026 (PMC5449643; doi:10.1093/gigascience/gix026)
Supplement: Reviewer_1_Report_(Original_Submission).pdf [file gix026_Reviewer_1_Report_(Original_Submission).pdf]

Reviewer Report

Title: "Genomic data for 78 chickens from 14 populations"

Version: Original Submission **Date:** 9/29/2016

Reviewer name: Martien Groenen

Reviewer Comments to Author:

Li et al describe an important, interesting data set of 78 individual chicken whole genome sequences. In that respect, the results and description of the data clearly warrant publication in GigaScience. However, the data and the results obtained from the population analyses done by the authors, are very poorly described. Moreover, the few conclusion drawn by the authors are not supported by the results of the analyses. Therefore, a major revision of the manuscript is essential before publication in GigaScience.

Major comments

The authors do not provide an extensive in depth analyses of the data and for the analyses that were done, the results are not very well presented. For example no information is provided for e.g.:

(1) Allele frequency spectrum for the identified SNPs

(2) Are there SNPs with high allele frequencies that are unique for a specific population/breed

(3) What are the allele frequencies for the unique new SNPs identified. I assume that many of these are very rare SNPs only seen in 1 individual (and the false discovery rate in this group is also likely to be higher).

(4) What is the total number of different SNPs identified?

(5) Do the SNP numbers only refer to single nucleotide substitutions, or did the authors also include small indels?

The authors describe that they generated a NJ tree and refer to figure 1. However, no tree is provided in figure 1. Also, the authors indicate that the PCA analysis confirms the seven distinct lineages (SW1-3, TC 1-3 and RJF). However this is not the case. In the PCA plot, SW2 is split into two separate clusters whereas one of these clusters completely overlaps with TC2.

The statement on page 4, lines 57-59 is not correct. Intergenic regions make up more that half of the chicken genome, so one would expect more than half of the SNPs to be located in this region.

Minor comments

Abstract, page 1, line 33; delete "with a total of 1.69T reads".

Abstract, page 1, line 36; "from 16 populations. In the supplementary table 1 I count 17 populations (Red Jungle Fowl samples are from Yunnan and from Hainan, so these should be considered as two separate populations).

Abstract, page 1, line 41. The comment "these data are abundant resources for poultry breeding". The data is important for population genetics and further studies in chicken and can contribute to breeding, but the statement as it is, is not correct.

Page 2, line 16: replace "alignment reads" by "aligned reads"

Findings, page 3 lines 13-23. This is repetitive and just a summary of methods and results.

Page 3, line14: "ancestry population". This is not correct as the ancestral population no longer exists (for that you need samples from > 10.000 years old.) This should be replaced by wild population, assumed to be representative of the original ancestral population.

Figure 1 and legends to figure 1: The abbreviations SW, TC are not clearly defined and that these refer to the eight lowland and six highland populations. I would suggest to add a table to the manuscript providing a summary of the populations used(separate columns for breed, group, and population number). In the legends the authors refer to nine domestic chicken in figure 1. However, in addition to the RJF I count 10.

Level of Interest

Please indicate how interesting you found the manuscript: An article whose findings are important to those with closely related research interests

Quality of Written English

Please indicate the quality of language in the manuscript: Acceptable

Declaration of Competing Interests

Please complete a declaration of competing interests, considering the following questions:

- Have you in the past five years received reimbursements, fees, funding, or salary from an organisation that may in any way gain or lose financially from the publication of this manuscript, either now or in the future?
- Do you hold any stocks or shares in an organisation that may in any way gain or lose financially from the publication of this manuscript, either now or in the future?
- Do you hold or are you currently applying for any patents relating to the content of the manuscript?
- Have you received reimbursements, fees, funding, or salary from an organization that holds or has applied for patents relating to the content of the manuscript?
- Do you have any other financial competing interests?
- Do you have any non-financial competing interests in relation to this paper?

If you can answer no to all of the above, write 'I declare that I have no competing interests' below. If your reply is yes to any, please give details below.

I declare that I have no competing interests'

I agree to the open peer review policy of the journal. I understand that my name will be included on my report to the authors and, if the manuscript is accepted for publication, my named report including any attachments I upload will be posted on the website along with the authors' responses. I agree for my report to be made available under an Open Access Creative Commons CC-BY license (http://creativecommons.org/licenses/by/4.0/). I understand that any comments which I do not wish to be included in my named report can be included as confidential comments to the editors, which will not be published.

I agree to the open peer review policy of the journal
