# Supplementary material for: Genomic data for 78 chickens from 14 populations
Source: Gigascience. 2017 Apr 18;6(6):1–5. doi: 10.1093/gigascience/gix026 (PMC5449643; doi:10.1093/gigascience/gix026)
Supplement: Reviewer_1_Report_(Revision_1).pdf [file gix026_Reviewer_1_Report_(Revision_1).pdf]

Reviewer Report

Title: "Genomic data for 78 chickens from 14 populations"

Version: Revision 1 **Date:** 11/18/2016

Reviewer name: Martien Groenen

Reviewer Comments to Author:

In their revised manuscript the authors have addressed many, but not all of my previous comments. Unfortunately, most of that information is buried in the supplementary material, and not discussed or referred to in the manuscript. The authors only discuss this in their reply to the comments of the reviewers. I think it is essential that the authors describe these issue in the paper itself. They should not only satisfy the reviewers, but should make the paper itself more clear to future readers.

(1) I think the authors have misunderstood my comment about the allele frequency spectra. I meant a plot that shows the number of SNPs for different MAF (minor allele frequency) classes. This is related to my previous comment number 3. Adding a plot for the SNPs that are already in dbSNP and the novel SNPs identified in this study should also show a clear difference (most novel ones have very low MAF).

(2) Related to the low MAF of the novel SNPs: I think the authors should comment on the false discovery rate of the SNPs or at least indicate that this is to be expected to be higher for SNPs with low MAF (so higher in the novel ones).

(3) I still do not agree with the grouping of the animals into 7 distinct clusters. The branches in the NJ tree are not very easy to see, but the tree nevertheless suggests that:

- SW2 and TC2 form 1 cluster consisting of 4 sub-clusters.

- Two of the RJF seem to cluster with SW3

- SW1 and TC3 cluster together into a single large cluster. If one would make separate sub-clusters, the TC3 individuals would be split into two subgroups together with different SW1 individuals.

This is also confirmed by the PCA plot, where SW2 and TC2 cluster together or form two separate clusters: (Cluster 1: 5 SW2 and 1 TC2 and cluster 2 with 6 SW2 and the remaining TC2 individuals. Likewise, SW1 and TC3 can be considered as 1 cluster (or two highly related populations with a bit of overlap).

(4) The abbreviations SW, TC are still not defined in the manuscript.

(5) The authors have added information about small indels to the supplement. However this is not mentioned in the manuscript. (also they still do not clearly mention in the manuscript that the numbers all refer to substitutions).

(6) The novel SNPs should be submitted to dbSNP and accession numbers should be provided in the manuscript.

Level of Interest

Please indicate how interesting you found the manuscript: An article whose findings are important to those with closely related research interests

Quality of Written English

Please indicate the quality of language in the manuscript: Needs some language corrections before being published

Declaration of Competing Interests

Please complete a declaration of competing interests, considering the following questions:

- Have you in the past five years received reimbursements, fees, funding, or salary from an organisation that may in any way gain or lose financially from the publication of this manuscript, either now or in the future?
- Do you hold any stocks or shares in an organisation that may in any way gain or lose financially from the publication of this manuscript, either now or in the future?
- Do you hold or are you currently applying for any patents relating to the content of the manuscript?
- Have you received reimbursements, fees, funding, or salary from an organization that holds or has applied for patents relating to the content of the manuscript?
- Do you have any other financial competing interests?
- Do you have any non-financial competing interests in relation to this paper?

If you can answer no to all of the above, write 'I declare that I have no competing interests' below. If your reply is yes to any, please give details below.

I declare that I have no competing interests

I agree to the open peer review policy of the journal. I understand that my name will be included on my report to the authors and, if the manuscript is accepted for publication, my named report including any attachments I upload will be posted on the website along with the authors' responses. I agree for my report to be made available under an Open Access Creative Commons CC-BY license (http://creativecommons.org/licenses/by/4.0/). I understand that any comments which I do not wish to be included in my named report can be included as confidential comments to the editors, which will not be published.

I agree to the open peer review policy of the journal
