# Supplementary material for: Genomic data for 78 chickens from 14 populations
Source: Gigascience. 2017 Apr 18;6(6):1–5. doi: 10.1093/gigascience/gix026 (PMC5449643; doi:10.1093/gigascience/gix026)
Supplement: reviewer_1_report_(Revision_2).pdf [file gix026_reviewer_1_report_(Revision_2).pdf]

**Reviewer Report**

**Title:** "Genomic data for 78 chickens from 14 populations"

**Version:** Revision 2 **Date:** 1/27/2017

**Reviewer name:** Martien Groenen

**Reviewer Comments to Author:**

In the revised manuscript the authors have sufficiently addressed most of my earlier comments. The only problem I still have is in the choice of the clustering (1-3) as suggested by the authors.In particular the grouping of the animals in cluster two seems odd when one looks at the result of the PCA analyses. I think it would make mores sense to split cluster 2 into two clusters. The first dimension of a PCA represents the largest difference seen between the individuals and cluster 2 actually consist of two clusters (2L and 2R) that are equally distant to each other as 2R is to cluster 3. I fail to see the arguments why the authors still group all of these into a single cluster.

A few minor comments:

the term overlapped SNPs seems a bit odd. I suggest to refer to these as "known SNPs". The contrast known vs novel seems more appropriate.

Line 144: (at least in an individual) replace "an" by "one"

I am confused by the "normalized depth" in line 145 (median of normalized depth of 1.14 versus 1.06). The authors state that the novel SNPs also exhibited a comparable sequencing depth with the overlapped SNPs (median of normalized depth of 1.14 versus 1.06). I probably misunderstand this term but could the authors explain what this means (given that the average sequencing depth is 18).

**Level of Interest**

Please indicate how interesting you found the manuscript: An article whose findings are important to those with closely related research interests

**Quality of Written English**

Please indicate the quality of language in the manuscript: Needs some language corrections before being published

**Declaration of Competing Interests**

Please complete a declaration of competing interests, considering the following questions:

- Have you in the past five years received reimbursements, fees, funding, or salary from an organisation that may in any way gain or lose financially from the publication of this manuscript, either now or in the future?
- Do you hold any stocks or shares in an organisation that may in any way gain or lose financially from the publication of this manuscript, either now or in the future?
- Do you hold or are you currently applying for any patents relating to the content of the manuscript?
- Have you received reimbursements, fees, funding, or salary from an organization that holds or has applied for patents relating to the content of the manuscript?
- Do you have any other financial competing interests?
- Do you have any non-financial competing interests in relation to this paper?

If you can answer no to all of the above, write 'I declare that I have no competing interests' below. If your reply is yes to any, please give details below.

I declare that I have no competing interests

I agree to the open peer review policy of the journal. I understand that my name will be included on my report to the authors and, if the manuscript is accepted for publication, my named report including any attachments I upload will be posted on the website along with the authors' responses. I agree for my report to be made available under an Open Access Creative Commons CC-BY license (http://creativecommons.org/licenses/by/4.0/). I understand that any comments which I do not wish to be included in my named report can be included as confidential comments to the editors, which will not be published.

I agree to the open peer review policy of the journal
