# Supplementary material for: Genomic data for 78 chickens from 14 populations
Source: Gigascience. 2017 Apr 18;6(6):1–5. doi: 10.1093/gigascience/gix026 (PMC5449643; doi:10.1093/gigascience/gix026)
Supplement: Reviewer_2_Report_(Original_Submission).pdf [file gix026_Reviewer_2_Report_(Original_Submission).pdf]

Reviewer Report

Title: "Genomic data for 78 chickens from 14 populations"

Version: Original Submission **Date:** 10/3/2016

Reviewer name: Andrew Mason

Reviewer Comments to Author:

The sequence data derived by the authors appears to be of high quality, with good coverage and support from multiple, individual data sets rather than the use of pools. This data will prove a good resource for researchers investigating breed-specific SNPs, as well as expanding the available non-commercial genome resources for the chicken.

In general the manuscript reads well, with clear methodology. My comments 1-8 are largely to do with sentence structure and consistency of abbreviations, rather than any strong issues with the science. I feel the manuscript would benefit from some more careful contextualisation of the results and some clear concluding remarks stressing the wider relevance of this data.

The additional file with this manuscript is clear and informative. At this stage I cannot seem to access the raw data on NCBI - the accession number is not found. Reviewer access would be appreciated, just to check the clarity of sample naming etc.

Specific comments (there were no line numbers, but the comments follow through the manuscript sequentially):

1. The sentence describing the filtering thresholds for read removal does not read well. Perhaps start with "Reads were removed if…" otherwise some of the filtering reasons are not in the correct tense.

2. The additional NGS data should be described in a separate paragraph to keep it separate from the sequencing the authors completed. It could remain in the same section, but it should be clearly separate.

3. The programs referenced in the three 'Data generation and analysis' sub-sections do not all have the program version used. These should be added in just as they have been for GATK and EIGENSOFT. I would also recommend having the web address for Picard as a reference, rather than in text.

4. The method for SNP calling is clear, but rather than simply stating the command parameters used, it would be useful to specifically state why their inclusion was necessary/required/recommended.

5. In the second sentence of 'Findings' explain the RJF abbreviation to the reader, then use the abbreviation consistently. In the next line you go back to using "red jungle fowl".

6. State explicitly that the wild RJF birds are being used as the "ancestry population".

7. As you have already defined the genome assembly and various acronyms in the 'Data Description' section, you do not need to do so again here. Additionally, if you wish to give the full name for BWA this should have been done in 'Data Description' section rather than here. I would also suggest that SNP should be defined in the subheading used in 'Data Description' rather than in 'Findings'.

8. Explain the abbreviation "TC" in terms of sampling locations.

9. You mention a phylogenetic analysis using NJ and that it is similar to the PCA data you show. However, I don't see the phylogenetic data in the figure? This is also not mentioned in the 'Data generation and analysis' section.

10. Are the differences in genetic diversity significantly different? The $\theta\omega$ values are effectively the same in the domestics, but different to the RJFs, but the $\theta\pi$ values are largely similar, with the RJF more like the TC than other domestics. Can you comment on this further?

11. You say that "changes in regulatory sites may have played a prominent role" - could you overlap your SNPs with known regulatory regions to confirm this? Or perhaps model how many SNPs you would expect in intergenic/coding regions and see if your proportions differ significantly? Approximately half the chicken genome is intergenic, so perhaps your data matches random SNP accumulation, rather than the importance of intergenic SNPs for breed diversification.

12. The abstract gives a conclusion about the use and scope of this data. A concluding remarks section should also be included in the main manuscript body to round off the data note and give the reader appropriate context.

Level of Interest

Please indicate how interesting you found the manuscript: An article of importance in its field

Quality of Written English

Please indicate the quality of language in the manuscript: Needs some language corrections before being published

Declaration of Competing Interests

Please complete a declaration of competing interests, considering the following questions:

- Have you in the past five years received reimbursements, fees, funding, or salary from an organisation that may in any way gain or lose financially from the publication of this manuscript, either now or in the future?
- Do you hold any stocks or shares in an organisation that may in any way gain or lose financially from the publication of this manuscript, either now or in the future?

- Do you hold or are you currently applying for any patents relating to the content of the manuscript?
- Have you received reimbursements, fees, funding, or salary from an organization that holds or has applied for patents relating to the content of the manuscript?
- Do you have any other financial competing interests?
- Do you have any non-financial competing interests in relation to this paper?

If you can answer no to all of the above, write 'I declare that I have no competing interests' below. If your reply is yes to any, please give details below.

I declare that I have no competing interests.

I agree to the open peer review policy of the journal. I understand that my name will be included on my report to the authors and, if the manuscript is accepted for publication, my named report including any attachments I upload will be posted on the website along with the authors' responses. I agree for my report to be made available under an Open Access Creative Commons CC-BY license (http://creativecommons.org/licenses/by/4.0/). I understand that any comments which I do not wish to be included in my named report can be included as confidential comments to the editors, which will not be published.

I agree to the open peer review policy of the journal
