# Supplementary material for: Genomic data for 78 chickens from 14 populations
Source: Gigascience. 2017 Apr 18;6(6):1–5. doi: 10.1093/gigascience/gix026 (PMC5449643; doi:10.1093/gigascience/gix026)
Supplement: Reviewer_3_Report_(Original_Submission).pdf [file gix026_Reviewer_3_Report_(Original_Submission).pdf]

Reviewer Report

Title: "Genomic data for 78 chickens from 14 populations"

Version: Original Submission **Date:** 10/10/2016

Reviewer name: Linnea Smeds

Reviewer Comments to Author:

The authors have sequenced 78 domestic chickens from several highland and lowland populations, and performed SNP analysis on this and on already published data from wild red jungle fowl and game fowl. It is an extensive data set that can be used for many different kinds of evolutionary and genetic analyses, although it's not possible to draw any conclusions from only the SNP results reported without further analysis.

Comments:

The title is not well formulated. "Genomic data for 78 chickens from 15 populations" would be better. But as I understand it, only 14 populations was sequenced in this study, and the 15th was downloaded from NCBI?

The line numbers in the manuscript pdf does not seem to correspond to the actual lines, but I will refer to the given line numbers closest to the line I mean.

=Data generation and analysis=
Page 2, line 13-18: Please include version numbers for BWA, SAMtools and PICARD.

Page 2, line 16: Should it be "mismatches ≥ 5"? (i.e. reads with 5 mismatches or more were removed)

Page 2, line 26: I find it strange that the picard commands "AddOrReplaceReadGroups" and "FixMateInformation" are described in such detail when the indel realigning (which in my opinion is a more important step) is just briefly mentioned. Which software was used for example? (I assume GATK RealignerTargetCreator and IndelRealigner? Please include version number as well).

=SNP calling=
Page 2, line 33-40: I found the following two descriptions very confusing: "To identify high-credibility variation in the 91 chickens, the highest-accuracy alignment was first processed [..]" and "We then detected genomic variants [..]". It sounds like SAMTools was used first to get sites with high credibility, and THEN that SNP calling was performed with GATK, but reading further (under "Findings") I understand that this was just two different ways of calling variants, and that the authors then use the intersect from the two results. Maybe this can be clarified already in this paragraph? Why were two different methods used in the first place? It is to my knowledge not common practice, neither is taking the intersect because the different programs might have different strengths and there will likely be true SNPs that one software picks up and the other miss, and vice versa. If the reason is to reduce the number of false positives maybe this can be elaborated on a bit further?

Page 2, line 35: Please include version number of SAMTools. In the current version, the parameters -S and -D are deprecated and the -C parameter should be given a value (integer).

Page 2, line 38: Is a minimum coverage of 4 enough to confidently call SNPs? For a heterozygous site covered by only four reads, the minor allele will have either one or two reads supporting it. Can one then be sure that a SNP is not just caused by a wrong base call? Or must the variant be seen in multiple individuals to count? If so what thresholds are used?

Page 2, line 43: Was there any particular reason for that JointGenotyper (which perform joint genotyping on a population basis) wasn't used after HaplotypeCaller, as recommended by GATK Best Practices?

Page 2, line 46: It says "After SNP calling, we applied variant quality recalibration [..]" but then the command for 'VariantFiltration' is stated, which is not for variant quality score recalibration ('VQSR'), but for so called 'hard-filtering' (only recommended by GATK if VQSR is not possible, for example if the sample size is too small or there are no true variants that can be used for recalibration - neither should be the case here). So my question is: was VQSR performed at all, and if no, why not? (And if yes - why was hard-filtering used as well?)

=Figure 1=
*I think the figure is informative but it's a bit hard to keep track on the different populations between the different parts of the figure - maybe the numbers (and colors) in B can be added to the pictures in A and the plot in D?

*Also, it's a bit confusing that there is a "Chengdu" mentioned in the description (line 42) when there is no such name in the actual figure. (From the supplement I understand that it corresponds to the breed "Tianfu black-bone fowl", but it should be explained here as well).

*I can't find the "Shimian caoke fowl" (which has a picture in A and a bar in D) on the map in B nor in the figure text - why is that? If it doesn't fit the PCA pattern it should be mentioned somewhere.

*The scale (Mb) is missing on the x-axis in D, and an s is missing from SNPs in "Novel SNP" (next to red box).

=Findings=
Page 4, line 55: "Particularly, more than half of the SNPs were detected in intergenic regions in each population, suggesting that changes at regulatory sites may have played a prominent role in diversifying selection of various chicken breeds." Isn't it expected that most of the SNPs are found in intergenic regions, since most of the genome is intergenic? And this doesn't have to mean that regulatory changes affect diversifying selection, since most of the SNPs found are just random and will have no affect at all! It seems like a hasty conclusion without evidence, and the sentence should be rephrased or removed.

Page 5. line 2: "15 domestic populations" - there are still only 14 populations in Fig1 B, (either add Shimian or explain why it's not included - and in that case change above to 14).

Level of Interest

Please indicate how interesting you found the manuscript: An article of limited interest

Quality of Written English

Please indicate the quality of language in the manuscript: Needs some language corrections before being published

Declaration of Competing Interests

Please complete a declaration of competing interests, considering the following questions:

- Have you in the past five years received reimbursements, fees, funding, or salary from an organisation that may in any way gain or lose financially from the publication of this manuscript, either now or in the future?
- Do you hold any stocks or shares in an organisation that may in any way gain or lose financially from the publication of this manuscript, either now or in the future?
- Do you hold or are you currently applying for any patents relating to the content of the manuscript?
- Have you received reimbursements, fees, funding, or salary from an organization that holds or has applied for patents relating to the content of the manuscript?
- Do you have any other financial competing interests?
- Do you have any non-financial competing interests in relation to this paper?

If you can answer no to all of the above, write 'I declare that I have no competing interests' below. If your reply is yes to any, please give details below.

I declare that I have no competing interests

I agree to the open peer review policy of the journal. I understand that my name will be included on my report to the authors and, if the manuscript is accepted for publication, my named report including any attachments I upload will be posted on the website along with the authors' responses. I agree for my report to be made available under an Open Access Creative Commons CC-BY license (http://creativecommons.org/licenses/by/4.0/). I understand that any comments which I do not wish to be included in my named report can be included as confidential comments to the editors, which will not be published.

I agree to the open peer review policy of the journal
