# Supplementary material for: Genomic data for 78 chickens from 14 populations
Source: Gigascience. 2017 Apr 18;6(6):1–5. doi: 10.1093/gigascience/gix026 (PMC5449643; doi:10.1093/gigascience/gix026)
Supplement: Reviewer_3_Report_(Revision_1).pdf [file gix026_Reviewer_3_Report_(Revision_1).pdf]

Reviewer Report

Title: "Genomic data for 78 chickens from 14 populations"

Version: Revision 1 **Date:** 11/18/2016

Reviewer name: Linnea Smeds

Reviewer Comments to Author:

The authors' response clarifies most of my original points.

Table 1 was a neat addition to figure 1, but now when it's clearer I'm not sure I understand the meaning of the dashed circles (around the populations) in Fig 1A anymore. In the original figure the circles corresponded to the group(s), but in the revised figure the label TC1 has been removed from the left-most circle, and from Table 1 I understand that those three populations (13, 14, 15) actually contain individuals from all three TC groups - so maybe this circle can be removed altogether?

The colors helped me a lot when connecting the different parts of the figure, but personally I think it would be even clearer if also the numbers in Fig 1A could be repeated, preferably also in Table 1 (as it is now one need to go via the Figure text to connect Fig 1A and Table 1).

The additional file now also contains figures, but I can't view two of them (in Table S2 and the lower one in Table S5), it looks like the links are broken. The top figure in Table S5 lacks text in the color legend. Also I think the figures should be given separate names (Figure S1, S2 etc) so they can be referred to properly in the text.

The new supplementary table S4 (referred to as "Allele frequency spectrum" both on page 4, line 124 and on page 6, line 189) doesn't seem to contain the Allele frequency spectrum but rather a summary of the types of SNPs for each individual.

Minor comments:

Page 1, line 28: change "gigabase (Gb) high quality data of" to "gigabases (Gb) of high quality data from"

Page 1, line 43: "base reseuencing data" should be "bases of resequencing data". On a personal note I don't think I've seen the word "trillion" in a scientific text before and I think I would prefer to write it as *10^12, but of course it's not wrong as it is now.

Page 2, line 58: change "by following steps" to "by the following steps"

Page 2, line 71: change "we further used command" to "we further used the command"

Page 4, line 123-124: the sentence "Among them, there was 1,812,591 (21.24%) SNPs could be detected" needs to be corrected, maybe remove "there was"?

Page 4, line 125: "was presented" -maybe use present tense when referring to table/figure?

Table S4: Header of column H should be changed from "No validated" to "Not validated".

Level of Interest

Please indicate how interesting you found the manuscript: An article whose findings are important to those with closely related research interests

Quality of Written English

Please indicate the quality of language in the manuscript: Not suitable for publication unless extensively edited

Declaration of Competing Interests

Please complete a declaration of competing interests, considering the following questions:

- Have you in the past five years received reimbursements, fees, funding, or salary from an organisation that may in any way gain or lose financially from the publication of this manuscript, either now or in the future?
- Do you hold any stocks or shares in an organisation that may in any way gain or lose financially from the publication of this manuscript, either now or in the future?
- Do you hold or are you currently applying for any patents relating to the content of the manuscript?

- Have you received reimbursements, fees, funding, or salary from an organization that holds or has applied for patents relating to the content of the manuscript?
- Do you have any other financial competing interests?
- Do you have any non-financial competing interests in relation to this paper?

If you can answer no to all of the above, write 'I declare that I have no competing interests' below. If your reply is yes to any, please give details below.

I declare that I have no competing interests.

I agree to the open peer review policy of the journal. I understand that my name will be included on my report to the authors and, if the manuscript is accepted for publication, my named report including any attachments I upload will be posted on the website along with the authors' responses. I agree for my report to be made available under an Open Access Creative Commons CC-BY license (http://creativecommons.org/licenses/by/4.0/). I understand that any comments which I do not wish to be included in my named report can be included as confidential comments to the editors, which will not be published.

I agree to the open peer review policy of the journal
